# Supplementary material for: Native yeast kinetochore structures identify an essential inner kinetochore interaction
Source: Nat Commun. 2026 Aug 5;17:9421. doi: 10.1038/s41467-026-76163-6 (PMC13538507; doi:10.1038/s41467-026-76163-6)
Supplement: Supplementary file 1 [file 41467_2026_76163_MOESM1_ESM.pdf]

Supplementary Material

1. Supplementary Figures
2. Supplementary Tables
3. Movie
4. References

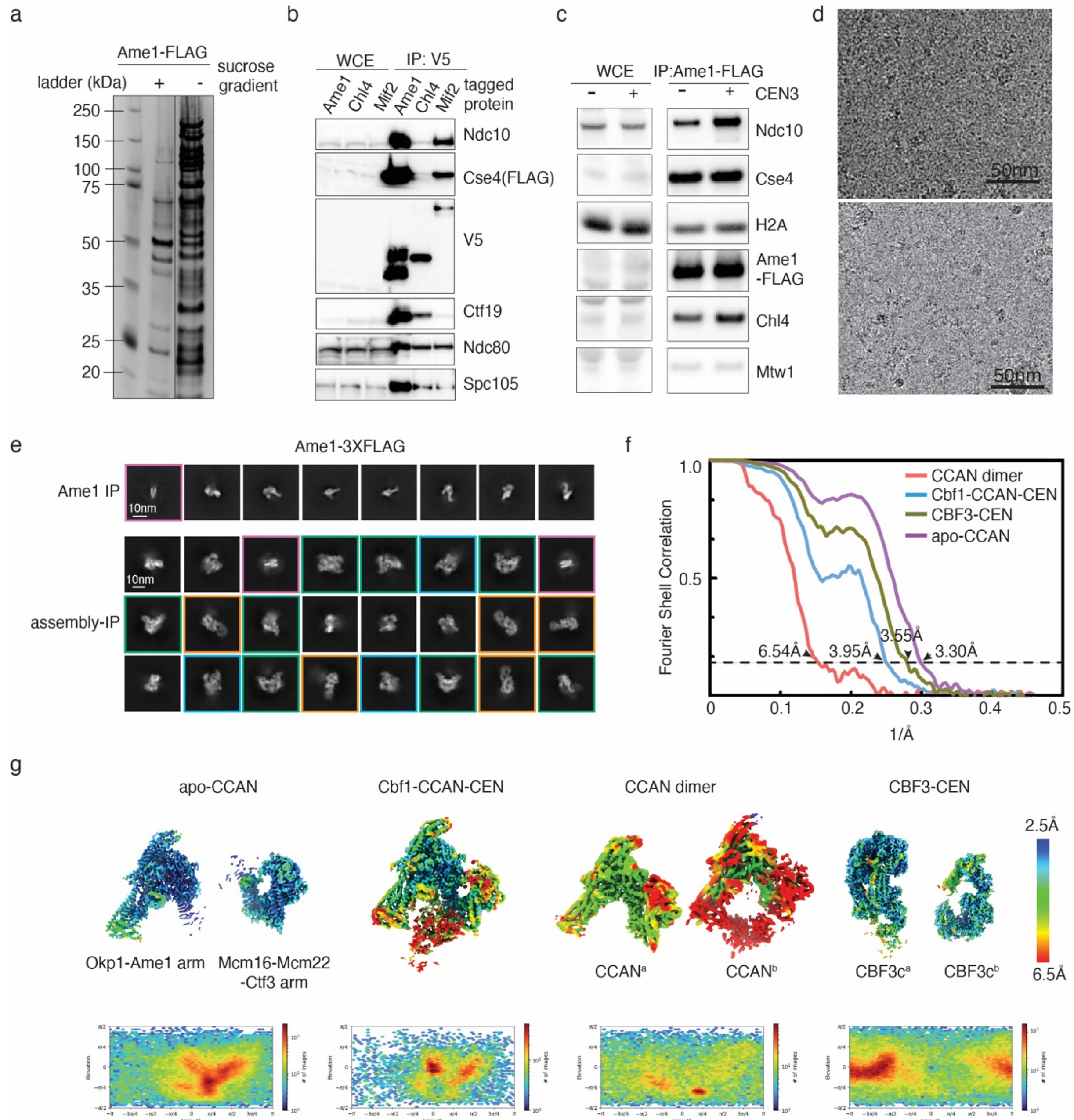

Supplementary Figure 1. Examination of eluates from Ame1-3XFLAG IP or assembly-IP using sucrose gradient precleared lysates.

a). Silver-stained analysis of SDS-PAGE on eluates from Ame1-3XFLAG (SBY21782) IP using precleared (sucrose gradient) or regular lysate. The two lanes were from the same gel but trimmed to remove the non-related lanes indicated by the black line separation. The experiment was repeated three times with similar results.

b). Immunoblot of immunoprecipitation from lysates that had different kinetochore protein tags (SBY21489*: CHL4-3xV5,* SBY21488*: MIF2-3xV5,* SBY21120*: AME1-3xV5*). The tagged protein is indicated, and multiple kinetochore proteins were detected by immunoblotting with the corresponding antibodies. WCE is whole cell

lysate. Source data are provided as a Source Data file.

c). Immunoblots show enrichment of kinetochore proteins from Ame1 IP or assembly IP using antibodies against the indicated kinetochore proteins. WCE is whole cell lysate. The experiment was repeated two times with similar results.

d). A representative micrograph of Ame1-3XFLAG IP eluate (upper panel) and assembly-IP eluate (lower panel).

e). 2D classification of Ame1-3XFLAG IP eluate (top row) or Ame1-3XFLAG assembly-IP eluate (bottom row). The averages of CCAN dimer are colored in green; the averages of Cbf1-CCAN-CEN are colored in blue; the averages of CBF3-CEN are colored in orange; the averages of nucleosome are colored in pink. The rest of 2D averages are apo-CCAN.

f). Overall resolutions were estimated with Fourier shell correlation (FSC) at 0.143 standard[1]. The local resolutions are shown by coloring densities with rainbow colors, with highest resolution at 2.5 Å colored in blue and the lowest resolution at 6.5 Å and colored in red. The name for each density is labeled.

g). Local resolution estimation of the four structures solved. Blue color represents higher resolution, and red color represents lower resolution. Angular distributions for each complex were shown in the bottom panel.

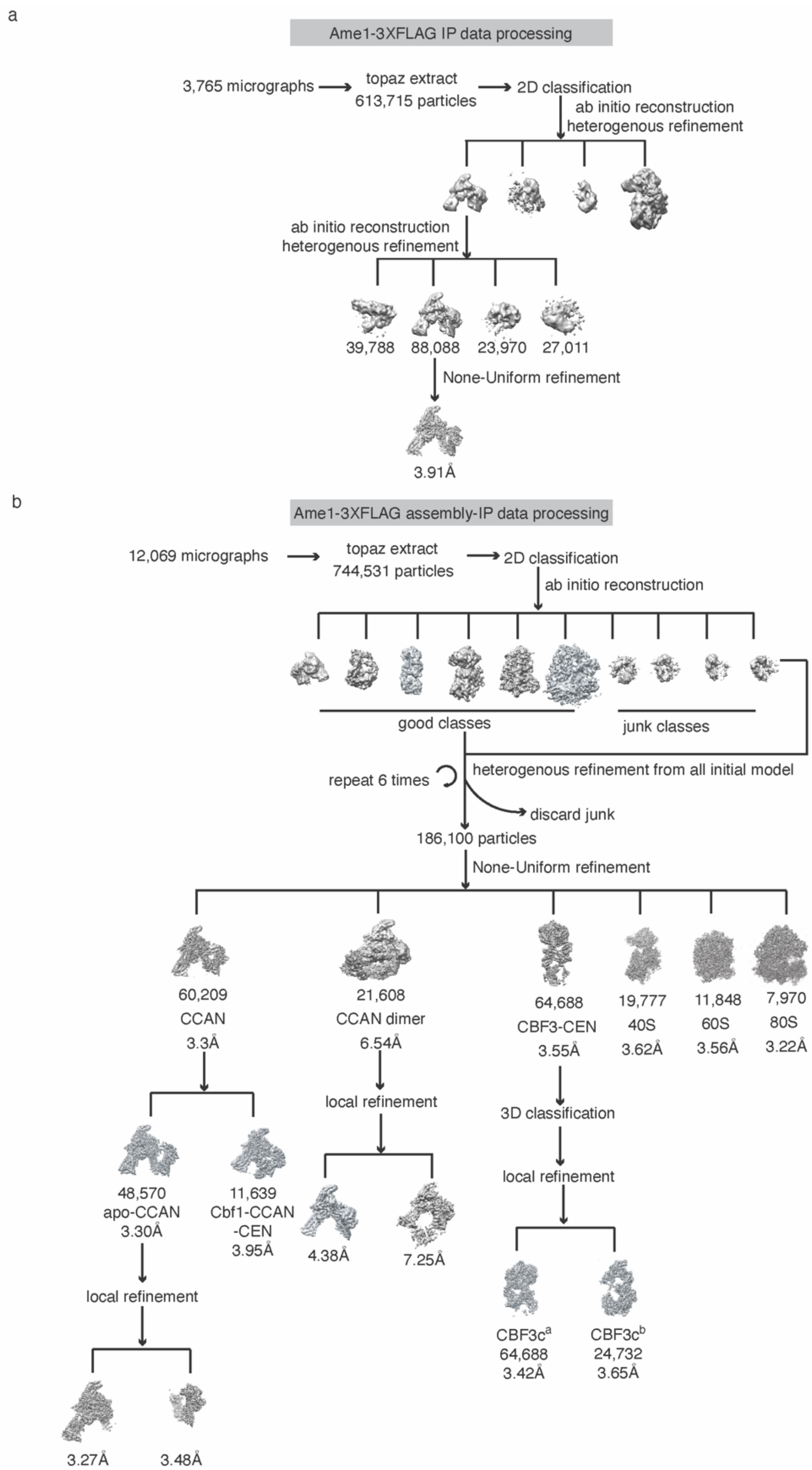

Supplementary Figure 2. Flowchart of cryoEM data processing and overall and local resolution of the structures identified from this work.

a). Data processing of Ame1-3XFLAG IP eluate.

b). Data processing of Ame1-3XFLAG assembly-IP eluate.

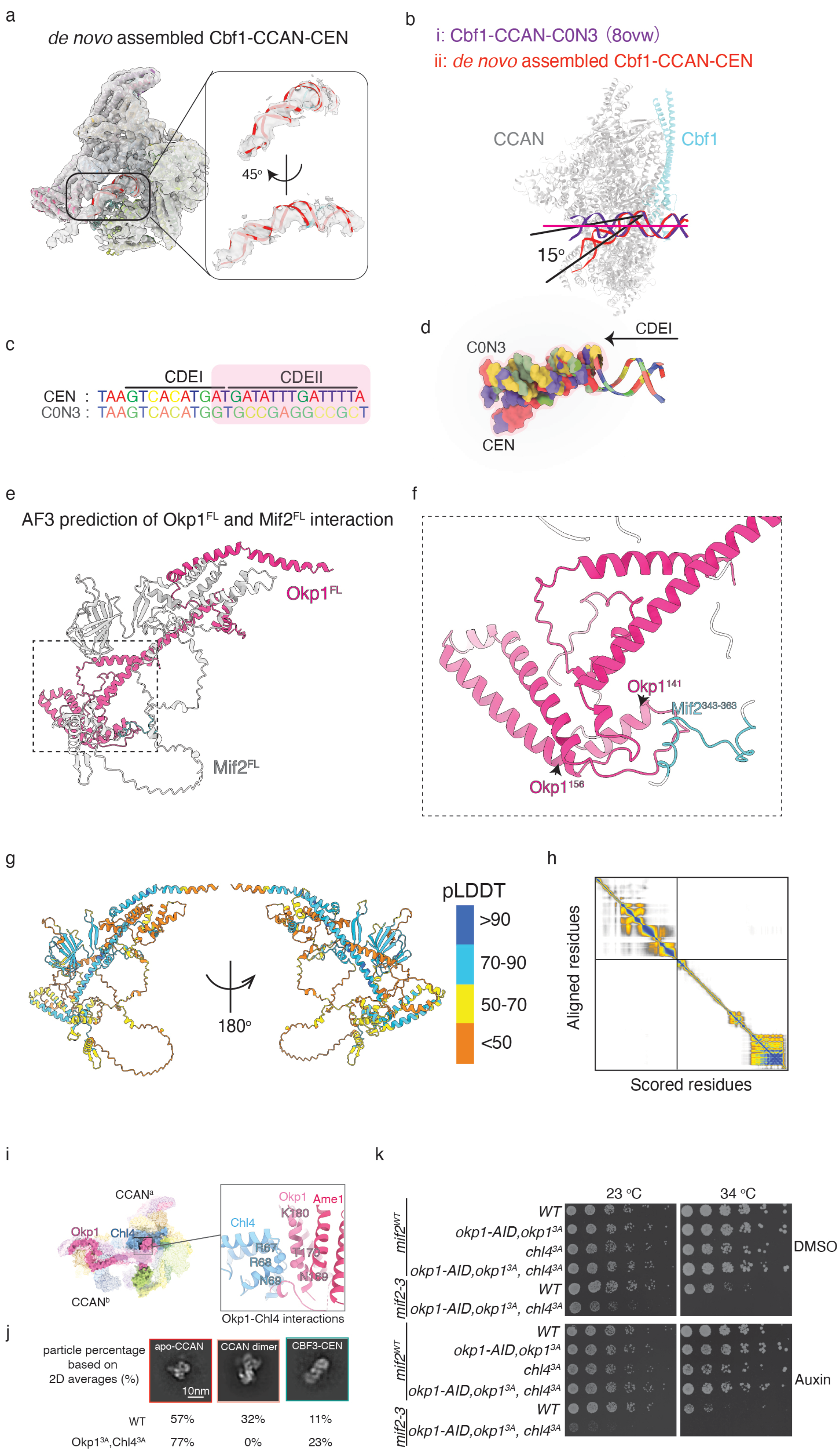
a
de novo assembled Cbf1-CCAN-CEN
45°
b
i: Cbf1-CCAN-C0N3 (8ovw)
ii: de novo assembled Cbf1-CCAN-CEN
CCAN
Cbf1
15°
c
CDEI
CDEII
CEN : TAAGTCACATGATGATATTTGATTTTA
C0N3 : TAAGTCACATGGTGCCGAGGCCGCT
d
CDEI
C0N3
CEN
e
AF3 prediction of Okp1FL and Mif2FL interaction
Okp1FL
Mif2FL
f
Okp1141
Mif2343-363
Okp1156
g
pLDDT
>90
70-90
50-70
<50
180°
h
Aligned residues
Scored residues
i
CCANa
Okp1
Chl4
CCANb
Chl4
Okp1
Ame1
K180
R67
R68
N69
T170
N169
Okp1-Chl4 interactions
j
particle percentage based on 2D averages (%)
apo-CCAN
CCAN dimer
CBF3-CEN
10nm
WT 57% 32% 11%
Okp13A,Chl43A 77% 0% 23%
k
23 °C
34 °C
mif2WT
WT
okp1-AID,okp13A
chl43A
okp1-AID,okp13A, chl43A
mif2-3
WT
okp1-AID,okp13A, chl43A
DMSO
mif2WT
WT
okp1-AID,okp13A
chl43A
okp1-AID,okp13A, chl43A
mif2-3
WT
okp1-AID,okp13A, chl43A
Auxin

Supplementary Figure 3. Comparison between native CCAN and recombinant reconstituted CCAN and DNA bending in the Cbf1-CCAN-CEN complex.

a). Model of *de novo* assembled Cbf1-CCAN-CEN fitted in low pass filtered density map to show the trace of DNA. The black box area is enlarged and cropped density of CEN DNA is shown. Two views were provided to show the DNA groove density.

b). Superimposition of the native Cbf1-CCAN-CEN and the reconstituted Cbf1-CCAN-C0N3 (8OVW)[2]. The model of CCAN is colored in transparent grey and Cbf1 is colored in cyan. CEN DNA is red and C0N3 DNA is purple. The angle difference between the two DNA molecules is demonstrated by a 15° angle.

c). Sequences of CEN and C0N3 DNA that interact with the CCAN-Cbf1 complex. The CDEI and part of the CDEII sequences are labeled. The sequence differences between CDEII are highlighted by transparent pink shade.

d). The two DNA sequences were superimposed with C0N3 transparent and CEN3 solid. The direction of DNA is labeled with arrows.

e). One AlphaFold model of proposed full-length (FL) Mif2 and Okp1 interaction. Okp1 is colored in hot pink and Mif2 is colored in grey.

f). Zoom-in view on the right, $Mif2^{343-363}$ fragment is colored in cyan and Okp1 is colored in pink.

g). Structure prediction of the $Okp1^{FL}$–$Mif2^{FL}$ complex was performed using AlphaFold Server, which uses AlphaFold 3. The top-ranked model was used for visualization and analysis. Model confidence was assessed using the pLDDT scores[3].

h). Predicted aligned error (PAE) plot for the AlphaFold 3 prediction of the Okp1–Mif2 complex. The x-axis represents the aligned residue, and the y-axis represents the scored residue. Black lines delineate the boundaries between the Okp1 and Mif2 chains.

i). Cartoon shows predicted interaction surface of CCAN dimer based on our EM density. Cα of residues that are involved in Okp1-Chl4 interface and Okp1-Ctf3 interface are shown. Okp1 is colored in pink and Chl4 is colored in blue.

j). Particle percentage of apo-CCAN, CCAN dimer and CBF3-CEN calculated from 2D classification using negative stain EM on WT or $okp1^{3A}chl4^{3A}$ mutant. In $okp1^{3A}chl4^{3A}$ mutant, no CCAN dimer is observed. At least 25,000 particles were analyzed for each of the WT and $okp1^{3A}chl4^{3A}$ mutant samples in a single independent experiment.

k). Viability assay using mutants on *chl4* and *okp1* under $23^{o}C$ or $34^{o}C$ are shown. The experiment was repeated three times with similar results.

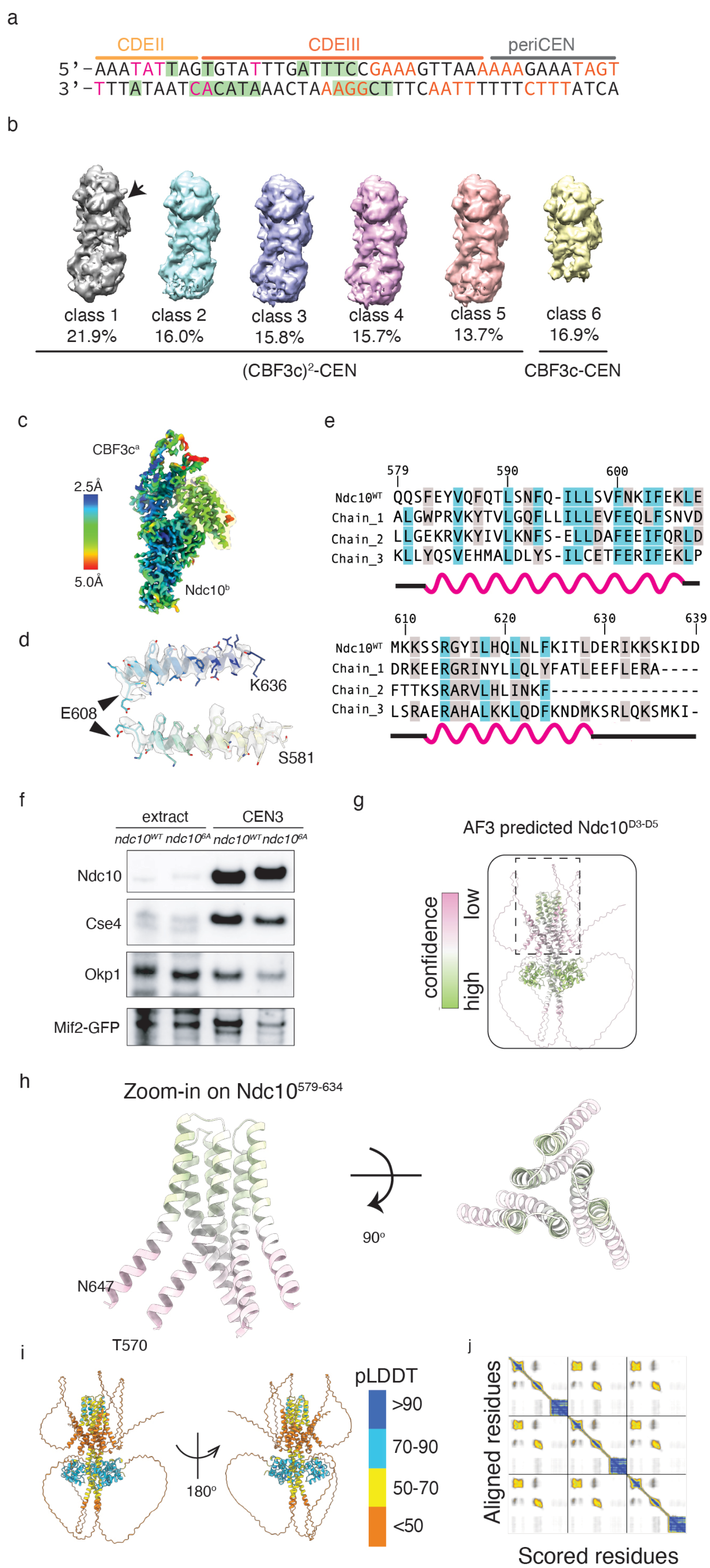
a
CDEII
CDEIII
periCEN
5'-AAATATTAGTGTATTTGATTTCCGAAAGTTAAAAAAGAAATAGT
3'-TTTATAATCACATAAACTAAAGGCTTTCAATTTTTTCTTTATCA
b
class 1
21.9%
class 2
16.0%
class 3
15.8%
class 4
15.7%
class 5
13.7%
class 6
16.9%
(CBF3c)²-CEN
CBF3c-CEN
c
CBF3cᵃ
2.5Å
5.0Å
Ndc10ᵇ
d
K636
E608
S581
e
579
590
600
Ndc10ᵂᵀ QQSFEYVQFQTLSNFQ-ILLSVFNKIFEKLE
Chain_1 ALGWPRVKYTVLGQFLLILLEVFEQLFSNVD
Chain_2 LLGEKRVKYIVLKNFS-ELLDAFEEIFQRLD
Chain_3 KLLYQSVEHMALDLYS-ILCETFERIFEKLP
610
620
630
639
Ndc10ᵂᵀ MKKSSRGYILHQLNLFKITLDERIKKSKIDD
Chain_1 DRKEERGRINYLLQLYFATLEEFLERA----
Chain_2 FTTKSRARVLHLINKF---------------
Chain_3 LSRAERAHALKKLQDFKNDMKSRLQKSMKI-
f
extract
CEN3
ndc10ᵂᵀ
ndc10⁶ᴬ
ndc10ᵂᵀ
ndc10⁶ᴬ
Ndc10
Cse4
Okp1
Mif2-GFP
g
AF3 predicted Ndc10ᴰ³⁻ᴰ⁵
confidence
low
high
h
Zoom-in on Ndc10⁵⁷⁹⁻⁶³⁴
90°
N647
T570
i
180°
pLDDT
>90
70-90
50-70
<50
j
Aligned residues
Scored residues

Supplementary Figure 4. Interaction between CBF3c and CEN3 DNA.

a). The regions of centromere determining elements CDEII, CDEIII and pericentromeric (periCEN) DNA that are protected by CBF3c are labeled. Base pairs that interact with Ndc10 and extra density are colored in orange and pink, respectively. Base pairs that interact with Cep3 are included in green shaded boxes.

b). 3D classification of all 6 classes of CBF3c-DNA particles. The arrow indicates the extra density. The ratio of each class is labeled. Class 6 is a CBF3c monomer-DNA complex.

c). Local resolution of focus refined density map. The high-resolution density is blue, and the low-resolution density is red. The color range is 2.5 Å to 5 Å.

d). Demonstration of model fitting into extra density with WT-Ndc10 sequence. The bulky side chain fitting is shown.

e). Sequence alignment for the predicted three chains aligned with WT-Ndc10. The highly conserved residues are blue, and the less conserved residues are grey. The secondary structure is shown in a cartoon.

f). Immunoblot of a kinetochore assembly assay on WT (SBY12796) or mutant *ndc10* (SBY23670) lysates. Source data are provided as a Source Data file. The experiment was repeated two times with similar results.

g). AlphaFold 3 (AF3) prediction of an Ndc10^{D3-D5} trimer. The Ndc10^{D3_trimer} is zoomed in and other regions were hidden.

h). Two zoom-in views from the boxed region in h).

i). pLDDT confidence of AlphaFold 3 model of the Ndc10^{D3-D5} trimer. Two orthogonal views of the top-ranked AlphaFold 3 model are shown, colored by per-residue predicted Local Distance Difference Test (pLDDT) score[3]. The confidence level is indicated by color key on the right.

j). Predicted aligned error (PAE) plot for Ndc10^{D3-D5} trimer. PAE is shown in Angstroms. The x-axis is scored residues, and the y-axis is aligned residues. Low PAE value is colored in blue, indicating high confidence in the relative positioning of residues and high PAE values colored in orange indicate low confidence, between different chains.

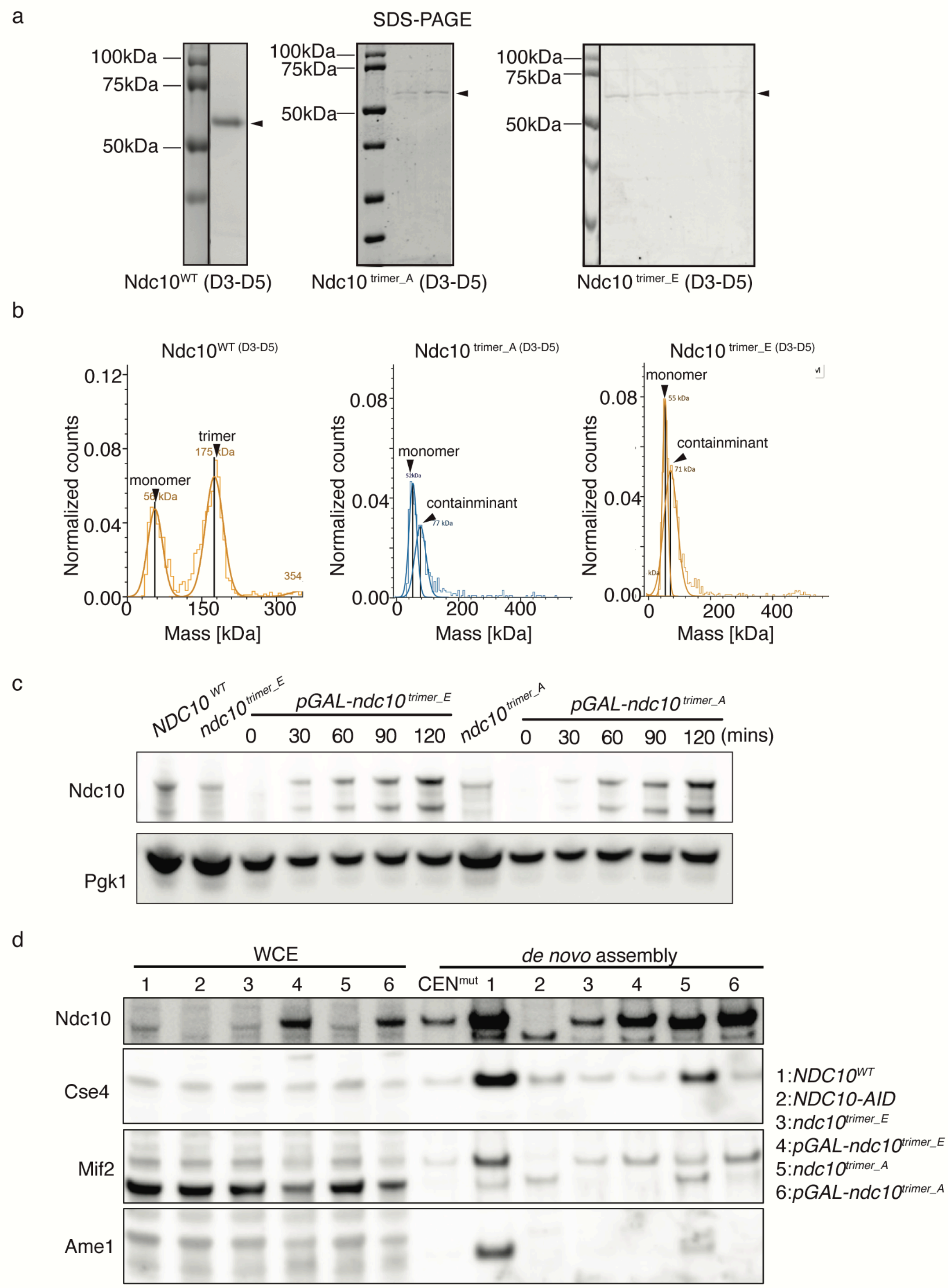

Supplementary Figure 5. Characterization of Ndc10 trimer proteins.

a). SDS-PAGE of purified WT-Ndc10, Ndc10 $^{trimer\_E}$ and Ndc10 $^{trimer\_A}$ proteins. The lanes in each box were from the same gel but trimmed to remove the non-related lanes indicated by a black line separation. Source data are provided as a Source Data file.

The experiment was repeated three times with similar results.

b). Mass photometry measurements of purified Ndc10(D3-D5)$^{trimer\_A}$ and Ndc10(D3-D5)$^{trimer\_E}$. The peak at ~55 kDa is an Ndc10 monomer and the peak at ~70 kDa is a contaminant protein. The experiment was repeated three times with similar results.

c). Immunoblot on whole cell extracts made from WT (SBY12796), *NDC10-AID* (SBY21327), *NDC10-AID ndc10*$^{trimer\_A}$ (SBY24533), *NDC10-AID ndc10*$^{trimer\_E}$ (SBY24478), *NDC10-AID pGAL-NDC10*^{WT} (SBY24690), *NDC10-AID pGAL-ndc10*$^{trimer\_A}$ (SBY24689), and *NDC10-AID pGAL-ndc10*$^{trimer\_E}$ (SBY24687) strains. Mutant protein overexpression was induced with 2% galactose, and samples removed every 30 minutes. The overexpression was observed starting at 30 minutes. The loading control is Pgk1. Source data are provided as a Source Data file. The experiment was repeated two times with similar results.

d). Immunoblot of kinetochore assembly assay on WT (SBY24587), *NDC10-AID* (SBY21327), *NDC10-AID ndc10*$^{trimer\_A}$ (SBY24533), *NDC10-AID ndc10*$^{trimer\_E}$ (SBY24478), *NDC10-AID pGAL-NDC10*^{WT} (SBY24690), *NDC10-AID pGAL-ndc10*$^{trimer\_A}$ (SBY24689), *NDC10-AID pGAL-ndc10*$^{trimer\_E}$ (SBY24687) extracts using antibodies against the indicated inner kinetochore protein. Source data are provided as a Source Data file. The experiment was repeated three times with similar results.

Table S1. Mass spectrometry result of Ame1-3XFLAG assembly-IP eluate.

Complex	Protein name	#PSMs
CBF3	Ctf13	12
	Cep3	24
	Ndc10	50
	Skp1	5
CCAN	Okp1	219
	Ctf19	80
	Ame1	112
	Mcm21	80
	Nkp1	140
	Nkp2	60
	Ctf3	8
	Mcm16	1
	Mcm22	11
	Chl4	19
	Iml3	38
	Cnn1	5
	Wip1	1
	Mif2	184
Chaperone	Scm3	4
Dam1c	Spc19	n.d.
	Dad1	3
	Spc34	n.d
	Dad2	n.d.
	Dad3	n.d.
	Dad4	n.d.
	Hsk3	n.d.
	Dam1	7
	Duo1	3
	Ask1	2
Kinase	Cdc5	14
	Mps1	63
	Pgk1	6
Knl1c	Kre28	5
	Spc105	10
Ndc80c	Nuf2	28
	Spc25	29
	Ndc80	36
	Spc24	39
Nucleosome	H2A	25
	H2B	31
	Cse4	94
	H4	38

#PSMs: Peptide Spectrum Matches

Table S2. Yeast strains used in this article

All strains are in the W303 strain background and available upon request.

Strain name	genotype	Used in Figure
SBY3	*MATa bar1-1 ade2-1 can1-100 his3-11 ura3-1 trp1-1 leu2-3,112*	Fig.5
SBY12796	*MATa NDC10-3XFLAG:KAN bar1-1 ade2-1 can1-100 his3-11 ura3-1 leu2-3,112 trp1-1*	Fig. 6,<br>Supplementary Fig. 4-5
SBY21120	*MATa AME1-3XV5:KAN leu2-3,112::pGAL-SCM3-mCherry:LEU2 ura3-1::CSE4-FLAG:URA3 cse4^:KAN ade2-1 bar1 trp1-1 his3-11 can1-100*	Supplementary Fig. 1
SBY21327	*MAT**α** NDC10-3XV5-IAA7:KAN leu2-3,112:: pGPD1-OsTIR1:LEU2 bar1-1 ade2-1 can1-100 his3-11 trp1-1 ura3-1*	Fig. 6,<br>Supplementary Fig.5
SBY21479	*MATa mif2-3 ura3-1 leu2-3,112 ade2-1 can1-100 his3-11 trp1-1 bar1-1*	Fig. 5
SBY21488	*MAT**α** MIF2-3XV5:HIS3 leu2-3,112::pGAL-SCM3-mCherry:LEU2 CSE4-FLAG:URA3 cse4^:KAN ade2-1 bar1 trp1-1 can1-100*	Supplementary Fig. 1
SBY21489	*MAT**α** CHL4-3XV5:HIS3 leu2-3,112::pGAL-SCM3-mCherry:LEU2 CSE4-FLAG:URA3 cse4^:KAN ade2-1 bar1 trp1-1 can1-100 his3-11*	Supplementary Fig. 1
SBY21782	*MAT**α** AME1-3XFLAG:TRP1 ura3-1 his3-11 trp1-1 leu2-3,112 ade2-1 can1-100 bar1*	Fig. 1,<br>Supplementary Fig.1
SBY22094	*MAT**α** MIF2-GFP:KAN bar1-1 ade2-1 can1-100 trp1-1 his3-11 leu2-3,112 ura3-1*	Fig. 5
SBY22191	*MAT**α** Ndc10-GFP:KAN bar1 ade2-1 can1-100 his3-11 ura3-1 leu2-3,112*	Fig. 4,<br>Supplementary Fig. 4
SBY23670	*MATa ndc10-S493A-D494A-S497A-D501A-H505A-K507A-3xFLAG:KAN bar1 ade2-1 can1-100 trp1-1 his3-11 leu2-3,112 ura3-1*	Fig. 5,<br>Supplementary Fig. 4

SBY23913	*MATα mif2-3 ndc10-S493A-D494A-S497A-D501A-H505A-K507A-3xFLAG:KAN bar1 ade2-1 can1-100 trp1-1 his3-11 leu2-3,112 ura3-1*	Fig. 5
SBY24238	*MATα MIF2-GFP:KAN ndc10-S493A-D494A-S497A-D501A-H505A-K507A-3xFLAG:KAN bar1 ade2-1 can1-100 trp1-1 his3-11 leu2-3,112 ura3-1*	Fig. 5
SBY24478	*MATα trp1-1::ndc10-F593E-L596E-F600E-F604E-3XV5:TRP1 NDC10-3XV5-IAA7:KAN leu2-3,112::pGPD1-OsTIR1:LEU2 bar1 ade2-1 can1-100 his3-11 ura3-1*	Fig. 6, Supplementary Fig.5
SBY24533	*MATα trp1-1::ndc10-F593A-L596A-F600A-F604A-3XV5:TRP1 NDC10-3XV5-IAA7:KAN leu2-3,112::pGPD1-OsTIR1:LEU2 bar1 ade2-1 can1-100 his3-11 ura3-1*	Fig. 6, Supplementary Fig.5
SBY24587	*MATα trp1-1::NDC10-3XV5:TRP1 NDC10-3XV5-IAA7:KAN leu2-3,112::pGPD1-OsTIR1:LEU2 bar1 ade2-1 can1-100 his3-11 ura3-1*	supplementary Fig.5
SBY24595	*MATa trp1-1::NDC10-3XV5:TRP1 NDC10-3XV5-IAA7:KAN leu2-3,112::pGPD1-OsTIR1:LEU2 MTW1-mYPet:URA3 SPC110-mTurquoise2:TRP1 bar1 ade2-1 can1-100 his3-11*	Fig. 6
SBY24687	*MATα trp1-1::pGAL-ndc10-F593E-L596E-F600E-F604E-3XV5:TRP1 NDC10-3XV5-IAA7:KAN leu2-3,112:: pGPD1-OsTIR1:LEU2 bar1 ade2-1 can1-100 his3-11 trp1-1 ura3-1*	Fig. 6, Supplementary Fig.5
SBY24689	*MATα trp1-1::pGAL-ndc10-F593A-L596A-F600A-F604A-3XV5:TRP1 NDC10-3XV5-IAA7:KAN leu2-3,112::pGPD1-OsTIR1:LEU2 bar1 ade2-1 can1-100 his3-11 trp1-ura3-1*	Fig. 6, Supplementary Fig.5
SBY24690	*MATa trp1-1::pGAL-NDC10-3XV5:TRP1 bar1 ade2-1 can1-100 his3-11 trp1-1 ura3-1 leu2-3,112*	Fig. 6, Supplementary Fig.5
SBY24691	*MATa trp1-1::ndc10-F593E-L596E-F600E-F604E-3XV5:TRP1 NDC10-3XV5-IAA7:KAN leu2-3,112::pGPD1-OsTIR1:LEU2 MTW1-mYPet:URA3 SPC110-mTurquoise2:TRP1 bar1 ade2-1 can1-100 his3-11*	Fig. 6
SBY24692	*MATa trp1-1::ndc10-F593A-L596A-F600A-F604A-3XV5:TRP1 NDC10-3XV5-IAA7:KAN leu2-3,112::pGPD1-OsTIR1:LEU2 MTW1-mYPet:URA3 SPC110-mTurquoise2:TRP1 bar1 ade2-1 can1-100 his3-11*	Fig. 6

Table S3. Plasmids used in this article

Plasmid	Description
pSB963	Plasmid containing pericentromere and centromere 3 to amplify 500 bp CEN3
pSB3218	pPGK1-SpCas9, GFP-sgRNA parent vector, *URA3* (A gift from Elçin Ünal)
pSB3562	*MTW1-3xmYPet*, *URA3* (*C. albicans*) (*MTW1* integrating, SnaBI)
pSB3658	6XHIS-Ndc10(D3-D5)-3XV5 for WT Ndc10(D3-D5) purification
pSB3674	Plasmid containing full-length *NDC10X3V5*, *TRP1*
pSB3675	Plasmid containing *ndc10*$^{trimer\_E}$*X3V5*, *TRP1*
pSB3673	Plasmid containing *okp1*^{3A}*-3HA*, *TRP1* for ectopic locus
pSB3681	Plasmid containing *ndc10*$^{trimer\_A}$*X3V5*, *TRP1*
pSB3695	Plasmid containing *pGAL-ndc10*$^{trimer\_E}$*X3V5*, *HIS3*
pSB3696	Plasmid containing *pGAL-ndc10*$^{trimer\_A}$*X3V5*, *HIS3*
pSB3730	6XHIS-Ndc10^{trimer_A}(D3-D5)-3XV5 for protein purification
pSB3731	6XHIS-Ndc10^{trimer_E}(D3-D5)-3XV5 for protein purification

Table S4. Data collection and refinement statistics

	apo-CCAN (EMD-75131) (PDB: 10FI)	Cbf1-CCAN-CEN (EMD-75095) (PDB: 10DQ)	CCAN dimer (EMD-75213) (PDB: 10JC)	CBF3-CEN (EMD-75107) (PDB: 10EH)
**Data collection and processing**				
Magnification	64,000	64,000	64,000	64,000
Voltage (kV)	300	300	300	300
Electron exposure (e–/Å^2)	50	50	50	50
Defocus range (μm)	-1~-2	-1~-2	-1~-2	-1~-2
Pixel size (Å)	1.07	1.07	1.07	1.07
Symmetry imposed	C1	C1	C1	C1
Final particle mages (no.)	48,570	11,639	21,608	64,688
Map resolution (Å) FSC threshold	3.30	3.95	6.54	3.55
**Refinement**				
Model resolution (Å) FSC threshold	3.30	3.95	6.54	3.55
Model composition				
Non-hydrogen atoms	18835	25868	47029	40188
Protein residues	2458	3190	5820	4611
DNA residues	0	54	0	88
R.m.s. deviations				
Bond lengths (Å)	0.004	0.003	0.004	0.008
Bond angles (°)	0.615	0.658	0.607	0.607
Validation				

MolProbity score	2.18	2.22	1.96	2.45
Clashscore	10.23	12.19	13.57	12.78
Poor rotamers (%)	2.51	2.95	0.00	4.05
Ramachandran plot				
Favored (%)	95.04	96.14	95.35	94.63
Allowed (%)	4.75	3.83	4.61	5.19
Disallowed (%)	0.21	0.03	0.04	0.18

Movie 1. Movement of CEN DNA when comparing native and reconstituted CBF3-CEN.

References

1. Henderson, R. *et al.* Outcome of the first electron microscopy validation task force meeting. *Structure* **20**, 205–214 (2012).

2. Dendooven, T., Zhang, Z., Yang, J., McLaughlin, S. H. & Schwab, J. Cryo-EM structure of the complete inner kinetochore of the budding yeast point centromere. *Sci. Adv.*

3. Abramson, J. *et al.* Accurate structure prediction of biomolecular interactions with AlphaFold 3. *Nature* **630**, 493–500 (2024).
