# Supplementary material for: Native yeast kinetochore structures identify an essential inner kinetochore interaction
Source: Nat Commun. 2026 Aug 5;17:9421. doi: 10.1038/s41467-026-76163-6 (PMC13538507; doi:10.1038/s41467-026-76163-6)
Supplement: Supplementary file 2 [file 41467_2026_76163_MOESM2_ESM.pdf]

Description of Additional supplementary files

Supplementary Movie 1: Movement of CEN DNA when comparing native and reconstituted CBF3- CEN.
