# Supplementary material for: Native yeast kinetochore structures identify an essential inner kinetochore interaction
Source: Nat Commun. 2026 Aug 5;17:9421. doi: 10.1038/s41467-026-76163-6 (PMC13538507; doi:10.1038/s41467-026-76163-6)
Supplement: Supplementary file 3 [file 41467_2026_76163_MOESM4_ESM.pdf]

nature portfolio

Corresponding author(s): Biggins, Sue

Last updated by author(s): 7/6/2026

Reporting Summary

Nature Portfolio wishes to improve the reproducibility of the work that we publish. This form provides structure for consistency and transparency in reporting. For further information on Nature Portfolio policies, see our Editorial Policies and the Editorial Policy Checklist.

Statistics

For all statistical analyses, confirm that the following items are present in the figure legend, table legend, main text, or Methods section.

n/a	Confirmed	
☐	☒	The exact sample size (*n*) for each experimental group/condition, given as a discrete number and unit of measurement
☐	☒	A statement on whether measurements were taken from distinct samples or whether the same sample was measured repeatedly
☐	☒	The statistical test(s) used AND whether they are one- or two-sided<br>*Only common tests should be described solely by name; describe more complex techniques in the Methods section.*
☒	☐	A description of all covariates tested
☒	☐	A description of any assumptions or corrections, such as tests of normality and adjustment for multiple comparisons
☐	☒	A full description of the statistical parameters including central tendency (e.g. means) or other basic estimates (e.g. regression coefficient) AND variation (e.g. standard deviation) or associated estimates of uncertainty (e.g. confidence intervals)
☐	☒	For null hypothesis testing, the test statistic (e.g. *F*, *t*, *r*) with confidence intervals, effect sizes, degrees of freedom and *P* value noted<br>*Give P values as exact values whenever suitable.*
☒	☐	For Bayesian analysis, information on the choice of priors and Markov chain Monte Carlo settings
☒	☐	For hierarchical and complex designs, identification of the appropriate level for tests and full reporting of outcomes
☒	☐	Estimates of effect sizes (e.g. Cohen's *d*, Pearson's *r*), indicating how they were calculated

Our web collection on statistics for biologists contains articles on many of the points above.

Software and code

Policy information about availability of computer code

Data collection	SerialEM was used for cryoEM data collection (https://bio3d.colorado.edu/SerialEM). Alpha Fold 3 server was used for inital model building (https://alphafoldserver.com/) and protein complex interaction prediction. Pacbio-subreads were used for Fiber-seq data collection (https://github.com/PacificBiosciences/PacBioFileFormats/blob/13.0/SubreadsBAM.rst). Mass photometry data was collected using Acquire2024 R1.1 (https://refeyn.com/software-release-updates#AcquireMP). SoftWorX v7.2.1 was used for DeltaVision data collection (https://download.cytivalifesciences.com/cellanalysis/download_data/softWoRx/7.2.1/).
Data analysis	Cryosparc4.0 (https://cryosparc.com) was used for cryoEM data analysis. Phenix 1.21.1(https://phenix-online.org/) and coot 0.9(https://www2.mrc-lmb.cam.ac.uk/personal/pemsley/coot/) were used for model building. ChimeraX 1.71.1 was used for generating figure (https://www.cgl.ucsf.edu/chimerax/). FIJI was used for TIRF data analysis (https://fiji.sc/). TIRF images were analyzed using CellProfiler (https://cellprofiler.org/). Custom software written in MATLAB (R2021a) was used for TIRF colocalization residence lifetime analysis and plot generation. The source code is publicly available at https://github.com/FredHutch/Automated-Single-Molecule-Colocalization-Analysis.

For manuscripts utilizing custom algorithms or software that are central to the research but not yet described in published literature, software must be made available to editors and reviewers. We strongly encourage code deposition in a community repository (e.g. GitHub). See the Nature Portfolio guidelines for submitting code & software for further information.

Data

Policy information about availability of data

All manuscripts must include a data availability statement. This statement should provide the following information, where applicable:

- Accession codes, unique identifiers, or web links for publicly available datasets
- A description of any restrictions on data availability
- For clinical datasets or third party data, please ensure that the statement adheres to our policy

Mass spectrometry data generated in this study is available through Mass Spectrometry Interactive Virtual Environment (MassIVE, University of California San Diego) with the link https://massive.ucsd.edu/ProteoSAFe/private-dataset.jsp?task=a50a708407634efc951e1828867bd934. The density map and model of apo-CCAN are accessible with code EMD-75131 from EM Databank and 10FI from Protein Data Bank. The density map and model of CCAN dimer are accessible with code EMD-75213 from EM Databank and 10JC from Protein Data Bank. The density map and model of Cbf1-CCAN-CEN are accessible with code EMD-75095 from EM Databank and 10DQ from Protein Data Bank. The density map and model of CBF3-CEN are accessible with code EMD-75107 from EM Databank and 10EH from Protein Data Bank. The plasmids and primers used in this study can be provided when requested.The Fiber-seq sequencing data generated in this study for the ndc10-6A mutant have been deposited in the NCBI Sequence Read Archive (SRA) database under the accession number PRJNA1427749. Fiber-seq data for the WT strain are available at SRA under accession number PRJNA1189155.

Research involving human participants, their data, or biological material

Policy information about studies with human participants or human data. See also policy information about sex, gender (identity/presentation), and sexual orientation and race, ethnicity and racism.

Reporting on sex and gender	*Use the terms sex (biological attribute) and gender (shaped by social and cultural circumstances) carefully in order to avoid confusing both terms. Indicate if findings apply to only one sex or gender; describe whether sex and gender were considered in study design; whether sex and/or gender was determined based on self-reporting or assigned and methods used.<br>Provide in the source data disaggregated sex and gender data, where this information has been collected, and if consent has been obtained for sharing of individual-level data; provide overall numbers in this Reporting Summary. Please state if this information has not been collected.<br>Report sex- and gender-based analyses where performed, justify reasons for lack of sex- and gender-based analysis.*
Reporting on race, ethnicity, or other socially relevant groupings	*Please specify the socially constructed or socially relevant categorization variable(s) used in your manuscript and explain why they were used. Please note that such variables should not be used as proxies for other socially constructed/relevant variables (for example, race or ethnicity should not be used as a proxy for socioeconomic status).<br>Provide clear definitions of the relevant terms used, how they were provided (by the participants/respondents, the researchers, or third parties), and the method(s) used to classify people into the different categories (e.g. self-report, census or administrative data, social media data, etc.)<br>Please provide details about how you controlled for confounding variables in your analyses.*
Population characteristics	*Describe the covariate-relevant population characteristics of the human research participants (e.g. age, genotypic information, past and current diagnosis and treatment categories). If you filled out the behavioural & social sciences study design questions and have nothing to add here, write "See above."*
Recruitment	*Describe how participants were recruited. Outline any potential self-selection bias or other biases that may be present and how these are likely to impact results.*
Ethics oversight	*Identify the organization(s) that approved the study protocol.*

Note that full information on the approval of the study protocol must also be provided in the manuscript.

Field-specific reporting

Please select the one below that is the best fit for your research. If you are not sure, read the appropriate sections before making your selection.

☒ Life sciences ☐ Behavioural & social sciences ☐ Ecological, evolutionary & environmental sciences

For a reference copy of the document with all sections, see nature.com/documents/nr-reporting-summary-flat.pdf

Life sciences study design

All studies must disclose on these points even when the disclosure is negative.

Sample size	CryoEM data: The number and identity of particles belong to each refined density map was identified through 3D classification and described in methods and Supplementary Figure 2.<br>TIRFM data: 3000 DNA molecules were imaged for each time point for 3 biological replicates resulting in a total of 9000 DNA molecules analyzed per time point.<br>For microscopy-based cell-counting assays, at least 60–200 cells were analyzed per biological replicate, with three independent biological replicates performed unless otherwise stated.
Data exclusions	CryoEM data: The CTF corrected micrographs with poor resolution limits and high defocus values were excluded in cryoSPARC and particles

Data exclusions	that were classified into good 3D density maps were excluded as they weaken the density signal.<br>Fluorescent microscopy data: When counting chromosome segregation, only cells that were clearly in anaphase were considered as data points.<br>No data were excluded from analysis.
Replication	All western blot analysis were repeated with two biological replicates.<br>All fluorescent microscopy data were repeated with three biological replicates that were used for statistics analysis.<br>All TIRF assays were repeated with three biological replicates and were all used for statistics analyses.<br>Three technical replicates were performed for mass photometry data.<br>cryoEM data: No replication was performed and all analysis algorithms were deterministic.
Randomization	For cryoEM data processing, the particle data was split randomly into two dataset and generated reconstructed density maps independently. The correlation between two density maps were evaluated following "gold-standard" protocol.<br>Randomization was not applicable for other studies.
Blinding	The investigators were not blinded to the allocation during experiments and outcome assessment.

Reporting for specific materials, systems and methods

We require information from authors about some types of materials, experimental systems and methods used in many studies. Here, indicate whether each material, system or method listed is relevant to your study. If you are not sure if a list item applies to your research, read the appropriate section before selecting a response.

Materials & experimental systems

n/a	Involved in the study
☐	☒ Antibodies
☒	☐ Eukaryotic cell lines
☒	☐ Palaeontology and archaeology
☒	☐ Animals and other organisms
☒	☐ Clinical data
☒	☐ Dual use research of concern
☒	☐ Plants

Methods

n/a	Involved in the study
☒	☐ ChIP-seq
☒	☐ Flow cytometry
☒	☐ MRI-based neuroimaging

Antibodies

Antibodies used	1.Anti-Mif2, generated by GenScript, used at 1:3000;<br>2.Anti-Ctf19, generated by GenScript, used at 1:5000;<br>3.Anti-Okp1, generated by GenScript, used at 1:5000;<br>4. Anti-Ame1, generated by GenScript, used at 1:5000;<br>5. Anti-Ndc10, generated by GenScript, used at 1:5000;<br>6. Anti-Chl4, generated by GenScript, used at 1:5000;<br>7. Anti-Mtw1, generated by GenScript, used at 1:5000;<br>8. Anti-Ndc80, gift from Arshad Desai, used at 1:5000;<br>9. Anti-Spc105, generated by GenScript, used at 1:5000;<br>10.Anti-Pgk1, invitrogen, 459250, used at 1:10,000;<br>11. Anti-H2A, invitrogen, Invitrogen 39235, used at 1:2000;<br>12. Anti-Cse4 antibody, DOI: 10.1016/S1534-5807(03)00322-8,<br>13. Anti-FLAG and anti-V5 were generated by GenScript, they are both used at 1:5000.<br>14. All secondary antibodies used in this study were purchased from GE Life Sciences. The catalog numbers are for goat anti- rat antibody conjugated to horseradishperoxidase (HRP) (catalog 31470, GE Life sciences, Marlborough MA) or a donkey α-rabbit antibody conjugated to HRP (catalog A16023, GE Life sciences, Marlborough MA).
Validation	1. Antibody was validated using yeast strains with endogenous tags on Mif2 to identify the protein via a migration shift.<br>2. Antibodies were validated by using yeast lysates lacking the Ctf19 protein to ensure the band disappeared.<br>3. Antibodies were validated using yeast strains with an endogenous Okp1 tag to confirm a shift in migration.<br>4. Antibodies were validated using yeast strains with an endogenous Ame1 tag to confirm a shift in migration.<br>5. Antibodies were validated using yeast strains with an endogenous Nd10 tag to confirm a shift in migration.<br>6. Antibodies were validated by using yeast lysates lacking the Chl4 protein to ensure the band disappeared.<br>7. Antibodies were validated using yeast strains with an endogenous Okp1 tag to confirm a shift in migration.<br>8. Anti-Ndc80 N-terminus antibody was generated in Arshad Desai's lab and has been validated using yeast strains where the endogenous Ndc80 protein was epitope tagged with another epitope to confirm that the protein shifted and that the bands matched<br>9. Antibodies were validated using yeast strains with an endogenous Spc105 tag to confirm a shift in migration.<br>10. Antibodies have been used extensively in the lab and validated in previous publication<br>11. Antibodies were validated using recombinant histones.<br>12. Antibodies were previously validated using recombinant Cse4 and tagged Cse4 yeast strains.<br>13. Antibodies were validated to recognize multiple FLag or V5 epitope-tagged proteins in yeast lysates.

14. Secondary antibodies have been used extensively in our laboratory and validated by the same methods as the primary antibodies as well as with negative controls lacking primary antibodies to confirm specificity.

Plants

Seed stocks

Report on the source of all seed stocks or other plant material used. If applicable, state the seed stock centre and catalogue number. If plant specimens were collected from the field, describe the collection location, date and sampling procedures.

Novel plant genotypes

Describe the methods by which all novel plant genotypes were produced. This includes those generated by transgenic approaches, gene editing, chemical/radiation-based mutagenesis and hybridization. For transgenic lines, describe the transformation method, the number of independent lines analyzed and the generation upon which experiments were performed. For gene-edited lines, describe the editor used, the endogenous sequence targeted for editing, the targeting guide RNA sequence (if applicable) and how the editor was applied.

Authentication

Describe any authentication procedures for each seed stock used or novel genotype generated. Describe any experiments used to assess the effect of a mutation and, where applicable, how potential secondary effects (e.g. second site T-DNA insertions, mosiacism, off-target gene editing) were examined.
