# Supplementary material for: Native yeast kinetochore structures identify an essential inner kinetochore interaction
Source: Nat Commun. 2026 Aug 5;17:9421. doi: 10.1038/s41467-026-76163-6 (PMC13538507; doi:10.1038/s41467-026-76163-6)
Supplement: Supplementary file 4 [file 41467_2026_76163_MOESM5_ESM.pdf]

nature portfolio

Peer Review File

Native yeast kinetochore structures identify an essential inner kinetochore interaction

Corresponding Author: Dr Sue Biggins

This file contains all reviewer reports in order by version, followed by all author rebuttals in order by version.

Version 0:

Reviewer comments:

Reviewer #1

(Remarks to the Author)
Jiang et al have examined early intermediates in east kinetochore assembly, using authentic proteins purified by Ame1-IP from yeast cells arrested in mitosis. After adding CEN DNA (embedded in a longer molecule that includes some pericentromeric regions) they describe 4 complexes, two of them with DNA and two without. These are: (1) the CTF19c aka CCAN (this review happens to loathe the CCAN acronym -- apologies); (2) a dimer of CTF19c; (3) the CEN DNA with Cbf1 and Ctf19c; (4) two CBF3s on CEN DNA. The first and last are the major species present. The finding on which they concentrate is a trimeric "extra density" in structure (4) that they convincingly identify as threefold association of the Ndc10 D3, previously identified as the likely oligomerization region. Mutational studies in yeast indicated that the interactions within this oligomerization domain and potentially some interactions with D1-D2 are important for function.

General comments:
(1) The paper is hard to read, even for someone who knows most of the components, because it seems to swallow whole the relevance of previous structures, many of which (perhaps most of the ones that contain DNA) may have reconstitution artifacts. Even the notion that the DNA must be pre-bent for nucleosome incorporation seems to this reader unproven. That statement is certainly true for in vitro attempts, but Ndc10 brings along Scm3, which will create a very high local concentration of Cse4:H4, and perhaps that's enough.
(2) Ame1-IP guarantees that many of the components in the mixture will contain Ctf19c/CCAN or will have dissociated from such larger complexes. Is there independent evidence that Ctf19c/CCAN associates with DNA before nucleosome assembly? The published DNA complexes with mammalian CCAN could readily be in vitro assemblies only partly related to the real thing. In other words, while nucleosome assembly probably needs help, this reviewer greatly doubts that the current images of that help reflect what goes on in a yeast nucleus.
(3) How do we know that the second CBF3 in complex (4) is not an assembly artifact? Why do the authors think that a second CBF3 bound non-specifically is a biologically real phenomenon? Doubt on that point doesn't invalidate the discovery that Ndc10 D3 seems to be a trimerization domain rather than a dimerization domain. The authors of ref 29 found evidence of more than one Ndc10 "dimer" bound downstream of the CBF3 core -- perhaps, like the heat-shock transcription factor, it's really a trimer.
(4) Although the proteins come directly from yeast cells, DNA binding is still an in vitro event, so what distinguishes complexes (3) and (4) from the "reconstituted" complexes with which the authors contrast them are potential PTMs (especially for Ctf19c/CCAN), possible small factors not previously identified (nor explicitly identified here), etc.

Comments on the individual complexes described in the MS:
(1) The apo-Ctf19c/CCAN validates previous structures from recombinant proteins; it could easily be slightly more "open" than the recombinants structures with which the authors compare it.
(2) This reviewer suspects that the Ctf19c/CCAN dimer is not a functional intermediate. A quite different dimer in ref 33 was used for structure determination but described by the authors as a useful artifact rather than as a physiologically relevant form.
(3) How does the interaction of Ctf19c/CCAN with Cbf1 compare with the interaction described by Barford's group?
(4) See comment (3) above.

Bottom line: the data in this paper are useful, and the authors are entitled to their biases about which structures are meaningful. This reviewer believes that the assembly description in the central paragraph on p.12 is probably wrong, but that

should not prevent publication. Authors who publish solid data (and this laboratory certainly qualifies) have earned the right to speculate a bit. My recommendation is to simplify the narrative, try to avoid relying on supplementary figures (most readers haven't the patience to look at them), and reduce the posturing about the one-step protocol, which is a very helpful addition to the methods described in the literature, but does bring with it the bias that association with Ame1 defines what will be most interesting.

NOTE: I was unable to download the "related MS", so if it contains information that would answer some of my questions or contradict some of my points, feel free to ignore those items.

Reviewer #2

(Remarks to the Author)
In the manuscript “Native yeast kinetochore structures identify an essential inner kinetochore interaction,” Jiang et al. describe several cryoEM structures of centromeric complexes from S. cerevisiae obtained directly from yeast. The authors lysed mitotically arrested yeast, digested the DNA, and subjected the lysate to a sucrose gradient. Centromeric components were enriched by immunoprecipitation using FLAG-Ame1 protein, and the pulled complexes were analyzed by cryoEM. In a parallel experiment, before immunoprecipitation, the authors added 500 bp DNA containing a specific yeast centromeric sequence and performed another set of cryoEM analyses on the assembled complexes, referring to these as “assembly IP.” In the IP without added DNA, the authors detected only the monomeric CCAN complex, which closely resembled the high-resolution structure previously reported using purified components. However, in the IP with added DNA, the authors observed a CCAN dimer with a different orientation than that reported for purified CCAN components. Interestingly, they did not see DNA in these complexes, although they concluded that DNA is necessary for assembly, as the dimer is absent in the IP without added DNA. They also observed Cbf1-CCAN bound to the CEN sequence with more pronouncedly bent DNA. Most notably, the CBF3 structure bound to CEN DNA revealed an extra density that the authors attribute to a trimer of the D3 domain of Ndc10. Previous literature had not shown that this part of Ndc10 could be involved in centromeric complex formation or that it directs trimerization (dimerization had been proposed previously). The authors used photobleaching, AF3, and mass photometry on purified Ndc10 D3 segment to confirm that the trimer observed in cryoEM is genuine.
To confirm the importance of the newly identified Ndc10 D3 trimer domain, they mutated six residues involved in its interaction with the Ndc10 D2 domain to alanines and found impaired yeast growth (in Mif2 conditional mutants) and reduced complex assembly in their IPs. Since they propose that the interaction of the D3 trimer with DNA stabilizes and bends DNA, they used Fiber-seq to assess DNA protection in assemblies made with wild-type and mutant Ndc10, finding that the wild type indeed protects more DNA.
Finally, to test the importance of trimerization itself, they further mutated four hydrophobic residues involved in stabilizing the Ndc10 D3 trimer to alanines or aspartic acids and observed that these mutations do not interfere with in vitro complex assembly but result in yeast that cannot properly segregate chromosomes. Based on their structures, which they interpret as different steps in kinetochore assembly, and AF3 modeling, they propose a new sequence of events during kinetochore assembly.

This is very thorough and comprehensive work that reveals new and important aspects of yeast kinetochore assembly. All experiments are conducted to the highest standards, and interpretation, bias and future steps are properly addressed and discussed throughout the paper.

Minor comments for authors to consider:
- Include micrographs (with dimension bar) in the supplementary material.
- It would be helpful to know if the authors have tried using any other DNA in their assemblies, in terms of sequence or length.
- Pg 8: Supplementary figure 6e should be Supplementary figure 6f.

Reviewer #3

(Remarks to the Author)
I co-reviewed this manuscript with one of the reviewers who provided the listed reports. This is part of the Nature Communications initiative to facilitate training in peer review and to provide appropriate recognition for Early Career Researchers who co-review manuscripts.

Reviewer #4

(Remarks to the Author)
The kinetochore is an important structure that forms on the centromere to ensure the faithful segregation of chromosomes. To understand its structure, many studies have been performed using recombinant proteins and in vitro reconstituted complexes. While this structural information is useful, much remains unknown about how the kinetochore is organized in cells. In this paper, the authors address the challenge of understanding the structure of the kinetochore in budding yeast cells. They developed a new method to purify the native inner kinetochore complex. Using this method, the authors purified several complexes and applied them to cryo-EM analysis. They identified four distinct inner kinetochore complexes, two of which had not been previously visualized. They discovered that Ndc10 forms a trimer that associates with centromeric DNA and the CBF3 complex. Using yeast genetics, the authors demonstrated that Ndc10 trimerization is essential for kinetochore formation.

Overall, this is an interesting observation. They identified native kinetochore complexes using a newly developed method. This method is very useful. It is also notable that they found differences in the structures of native complexes from reported structures using reconstituted complexes. Notably, Ndc10 trimerization was absent from the reconstituted complexes. In addition to structural data, they conducted comprehensive studies to reach their conclusions. The data quality is relatively high, and the manuscript is suitable for publication in Nature Communications. I raised several points in the hope that they would be useful for improving the paper's quality further.

1. Although the authors ultimately focused on Ndc10 trimerization, they made other interesting observations. For example, the structure of the CCAN dimer is interesting, and I am curious about its biological significance. Based on their interpretation in the paper, the interaction between Mif2 (343-363) and Okp1 (132-152) may be involved in the formation of the CCAN dimer in coordination with DNA. I am uncertain about such a structure. It would be helpful if the authors proposed a structural model using AF3. Additionally, it would be helpful if they could show mutant data for Mif2 (343-363) or Okp1 (132-152).
2. The authors propose that the binding of CBF3 or Ndc10 to DNA facilitates centromeric nucleosome assembly by bending the centromeric DNA. Although this is an attractive model, I feel it is a slight overstatement. I am unsure if they have data showing that mutations in the DNA-binding sites of the CBF3 complex or Ndc10 cause assembly defects in the Cse4 nucleosome. If they had such data, it would support the model. If not, however, they should tone down the model. I am also curious about the cellular phenotype of yeast with Ndc10 two lysine mutations and loop 194-208 mutations (DNA-binding mutations).
3. Related to comment #2. They could not observe Cse4 in their structures. This may be due to the flexible structure of the Cse4 nucleosome in cells. However, since they cannot see Cse4 in their structure, the final model in Figure 5H is too strong.
4. The Ndc10 (6A) mutant is interesting because it shows synthetic lethality with an Mif2 mutant. However, there is no evidence that the mutant exhibits binding defects with another Nud10. It would be good if they showed some data for this.
5. Regarding comment #4, the existence of Ndc10 in cells is unclear (Figure 5h). Figure 5h shows the Ndc10 trimer domain. It is assumed that full Ndc10 forms a trimer via the trimer domain. Does this mean that if the Ndc10 trimer binds to another Ndc10, it may form a tetramer? Please clarify this point. It would be better if they showed some suggestive data for tetramer formation in cells.

Version 1:

Reviewer comments:

Reviewer #1

(Remarks to the Author)
The revised MS has met most of my concerns. While my own intuition is that DNA bending is not a "big deal" and that the function of the Ndc10 oligomerization element (clearly trimerization here) has to do with other aspects of the early assembly pathway, I have no objection to the speculations offerred by Jiang et al.

Whle reading carefully the revised version, I realized that there is a question I should have raised earlier (and that should NOT delay acceptance and publication). The second CBF3core, seen here and in 6GYS, could easily be an in vitro addition, recruited by Ndc10b (and by the extra DNA). In the (admittedly very early, with less fancy methods that we now have) crosslinking and footprinting work of Espelin et al, there was evidence of additional Ndc10 to the "right" of the well-bound two, but no evidence of an additional CBF3 core. Likewise, the fluorescence counting numbers from Joglekar indicate one, not two CBF3 cores per kinetochore (one Cep3 dimer), but more than one Ndc10 dimer (consistent with a single trimer, although the number in their metaphase occupancy table was 4, not 3). The present authors mention (almost parenthetically) the possibility of an "experimental artifact" yielding the second CBF3 core, but another possibility is that it is a transient occupancy of the early intermediates studied here.

Reviewer #4

(Remarks to the Author)
This is a revised version of the manuscript. They found that Ndc10 forms a trimer that binds to centromeric DNA and the CBF3 complex. While I thought the previous version was well-written, the paper has improved with the addition of data and revisions. I believe the authors addressed the reviewers' concerns. Therefore, I recommend accepting this manuscript for publication.

Open Access This Peer Review File is licensed under a Creative Commons Attribution 4.0 International License, which permits use, sharing, adaptation, distribution and reproduction in any medium or format, as long as you give appropriate credit to the original author(s) and the source, provide a link to the Creative Commons license, and indicate if changes were made.
In cases where reviewers are anonymous, credit should be given to 'Anonymous Referee' and the source.
The images or other third party material in this Peer Review File are included in the article's Creative Commons license, unless indicated otherwise in a credit line to the material. If material is not included in the article's Creative Commons license and your intended use is not permitted by statutory regulation or exceeds the permitted use, you will need to obtain permission directly from the copyright holder.
To view a copy of this license, visit https://creativecommons.org/licenses/by/4.0/

We are grateful to the reviewers for their thoughtful comments and suggestions. We have responded to each of them below:

Reviewer's Comments:

Reviewer #1 (Remarks to the Author)

Jiang et al have examined early intermediates in east kinetochore assembly, using authentic proteins purified by Ame1-IP from yeast cells arrested in mitosis. After adding CEN DNA (embedded in a longer molecule that includes some pericentromeric regions) they describe 4 complexes, two of them with DNA and two without. These are: (1) the CTF19c aka CCAN (this review happens to loathe the CCAN acronym -- apologies); (2) a dimer of CTF19c; (3) the CEN DNA with Cbf1 and Ctf19c; (4) two CBF3s on CEN DNA. The first and last are the major species present. The finding on which they concentrate is a trimeric "extra density" in structure (4) that they convincingly identify as threefold association of the Ndc10 D3, previously identified as the likely oligomerization region. Mutational studies in yeast indicated that the interactions within this oligomerization domain and potentially some interactions with D1-D2 are important for function.

General comments:
(1) The paper is hard to read, even for someone who knows most of the components, because it seems to swallow whole the relevance of previous structures, many of which (perhaps most of the ones that contain DNA) may have reconstitution artifacts. Even the notion that the DNA must be pre-bent for nucleosome incorporation seems to this reader unproven. That statement is certainly true for in vitro attempts, but Ndc10 brings along Scm3, which will create a very high local concentration of Cse4:H4, and perhaps that's enough.

Response: We have revised the text throughout the entire manuscript to better credit the prior recombinant work as well as to frame our work as complementary to the existing structures. Regarding the question of whether DNA must be pre-bent for nucleosome formation, we agree that it is not clear that centromeric DNA must be pre-bent for a nucleosome to form. It is an excellent point that a high local concentration of Cse4–H4 by Scm3 and Ndc10 could overcome the intrinsic stiffness of CDEII. We have therefore softened our wording in the Introduction to describe DNA bending as facilitating nucleosome assembly rather than a strict prerequisite. We also now explicitly state that the Scm3–Cse4–H4 delivery pathway may be sufficient for nucleosome formation (p. 12, paragraph 3). Our intention is not to argue that bending precedes nucleosome formation, but that the native CBF3- and Ndc10-dependent bends we observe represent one plausible means to overcome the poor nucleosome-forming properties of CDEII.

(2) Ame1-IP guarantees that many of the components in the mixture will contain Ctf19c/CCAN or will have dissociated from such larger complexes. Is there independent

evidence that Ctf19c/CCAN associates with DNA before nucleosome assembly? The published DNA complexes with mammalian CCAN could readily be in vitro assemblies only partly related to the real thing. In other words, while nucleosome assembly probably needs help, this reviewer greatly doubts that the current images of that help reflect what goes on in a yeast nucleus.

Response: We did not mean to imply that the Ctf19/CCAN associates with DNA prior to nucleosome assembly. We previously analyzed the kinetics of CCAN arrival on CEN DNA using a TIRF assay and it arrives later than Cse4. We also showed that the CCAN components Okp1/Ame1 stabilize the nucleosome after Cse4 arrival[1]. We have clarified the CCAN discussion in the revised manuscript to avoid implying it arrives early (P.3, paragraph 3; P. 12, paragraph 3; P.13, paragraph 2).

(3) How do we know that the second CBF3 in complex (4) is not an assembly artifact? Why do the authors think that a second CBF3 bound non-specifically is a biologically real phenomenon? Doubt on that point doesn't invalidate the discovery that Ndc10 D3 seems to be a trimerization domain rather than a dimerization domain. The authors of ref 29 found evidence of more than one Ndc10 "dimer" bound downstream of the CBF3 core -- perhaps, like the heat-shock transcription factor, it's really a trimer.

Response: We cannot rule out that the second CBF3 is an assembly artifact. Data quantifying the CBF3 complex *in vivo* has reported one copy in one paper[2] and two in another[3], so it is not clear how many copies are at endogenous kinetochores. To address this, we performed photobleaching on GFP tagged Ctf13 or Cep3 using the TIRF assay described in our manuscript. The result is shown below. We found that the recruitment of Cep3 or Ctf13 stabilized around 30 minutes assembly. The photobleaching steps of Ctf13 and Cep3 are 1.5 and 2.8, indicating a mixture of one and two copies of CBF3 in the *de novo* assembly assay. We therefore cannot draw a solid conclusion about the number of CBF3 complexes and have just proposed that structures containing two CBF3 might be intermediates in the revised text (p. 12, paragraph 3).

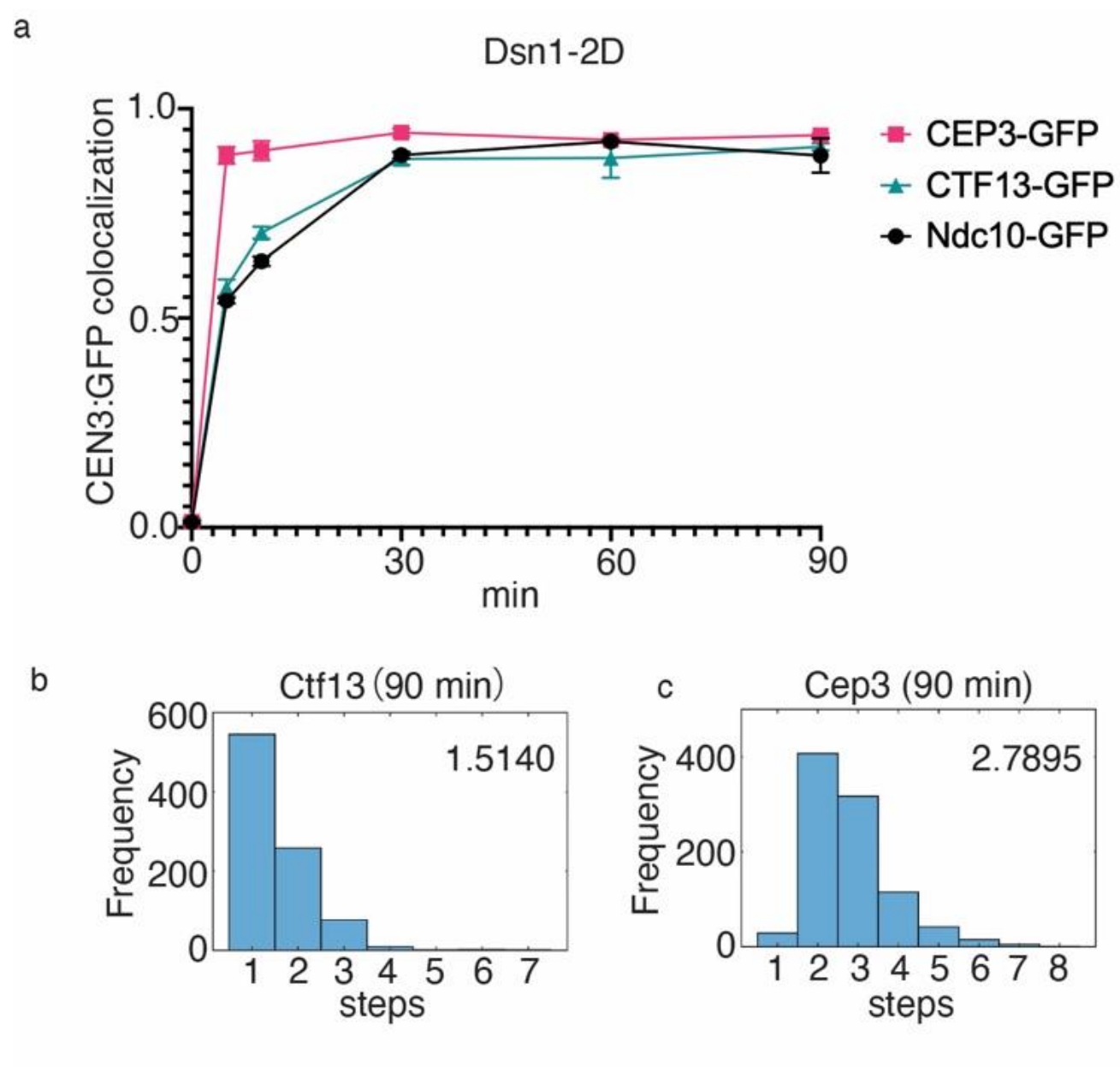

(4) Although the proteins come directly from yeast cells, DNA binding is still an in vitro event, so what distinguishes complexes (3) and (4) from the "reconstituted" complexes with which the authors contrast them are potential PTMs (especially for Ctf19c/CCAN), possible small factors not previously identified (nor explicitly identified here), etc.

Response: We agree and have now pointed out that our work builds on reconstituted structures by using native components assembled on DNA in the revised manuscript (p 4, paragraph 3).

Comments on the individual complexes described in the MS:
(1) The apo-Ctf19c/CCAN validates previous structures from recombinant proteins; it could easily be slightly more "open" than the recombinants structures with which the authors compare it.

Response: We agree that the apo-Ctf19/CCAN structure is very similar to the recombinant protein structure. In the revised text, we have eliminated the description of this structure and just stated that it is similar to previously published structures to make the manuscript easier to follow (p. 6, paragraph 1).

(2) This reviewer suspects that the Ctf19c/CCAN dimer is not a functional intermediate. A quite different dimer in ref 33 was used for structure determination but described by the authors as a useful artifact rather than as a physiologically relevant form.

Response: We were also unsure whether the CCAN dimer has any physiological function. To address this, we made a mutant that eliminates dimer formation. As expected since the CCAN is non-essential, the mutant cells are viable. However, we did detect a genetic interaction with a *mif2-3* temperature sensitive mutant (shown below), suggesting that the dimer does contribute to inner kinetochore function. We added this

data to Supplementary Fig. 3 but have also tempered the discussion of the dimer in the revised text.

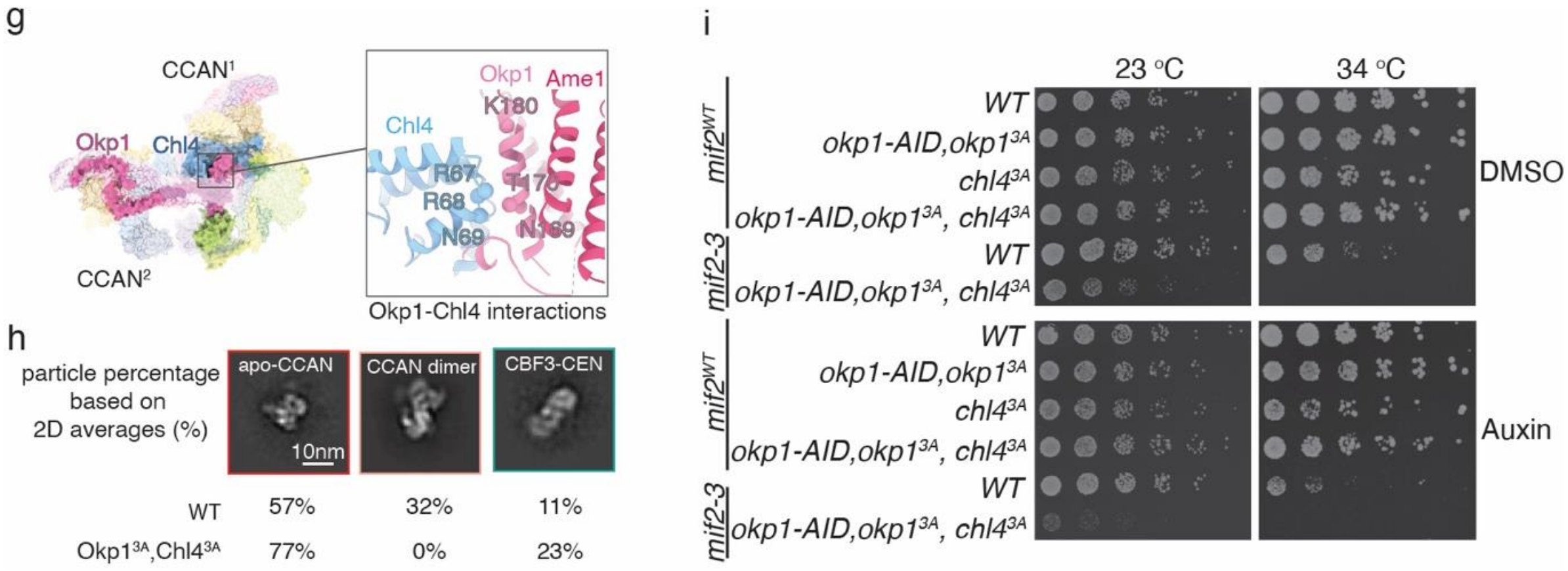

(3) How does the interaction of Ctf19c/CCAN with Cbf1 compare with the interaction described by Barford's group?

Response: We compared the structures and found that the RMSD between them is 0.405 after deleting the DNA molecule, which indicates these two Cbf1-CCAN structures are extremely similar. Specifically, the interaction between Cbf1 and Ctf19c/CCAN is the same so we have added this comparison to the figure shown below. The left shows the CCAN-Cbf1 interaction using our structure and the right shows a figure from Barford paper[4].

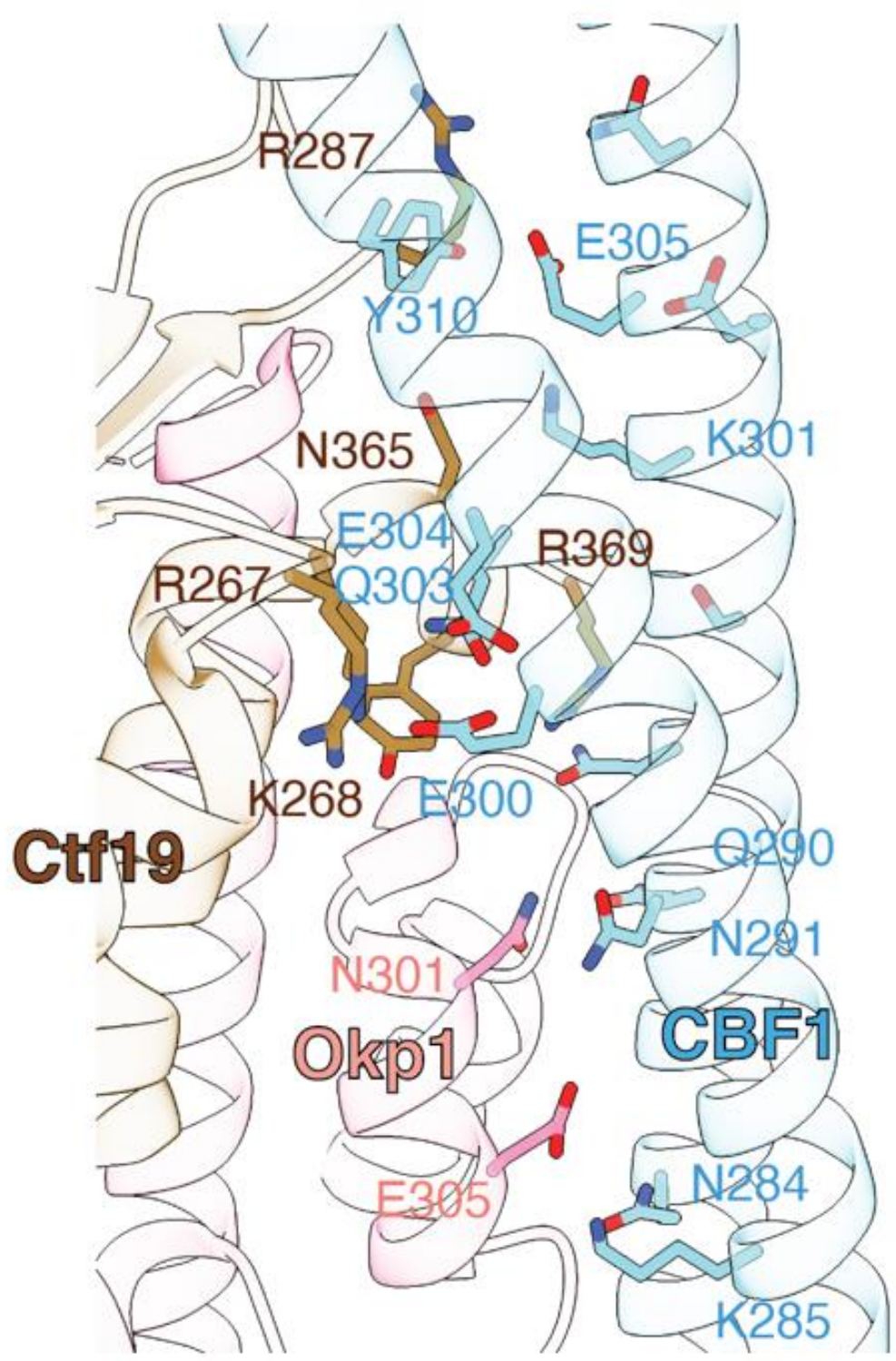

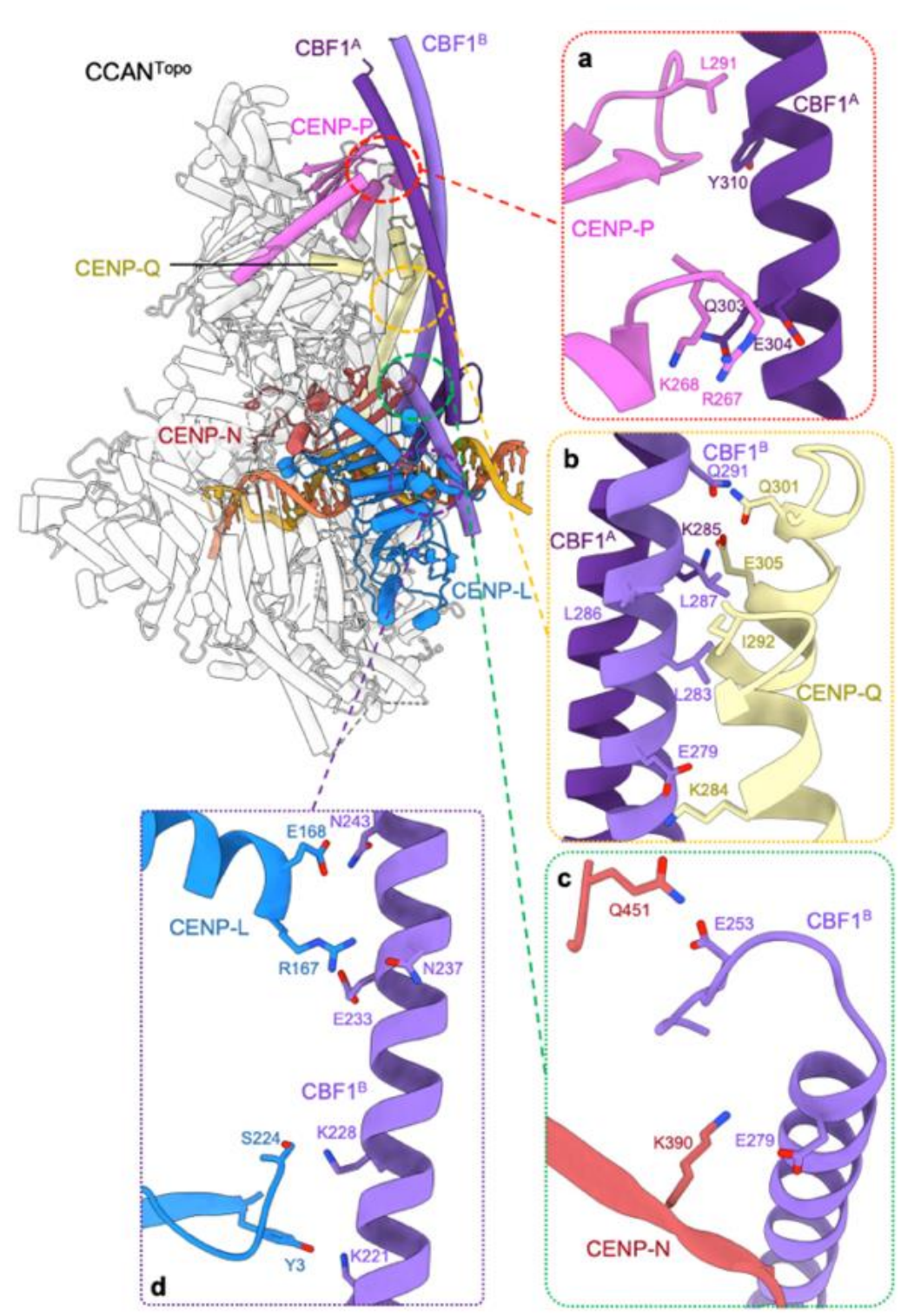

(4) See comment (3) above.

Response: We addressed the comments in (3) above.

Bottom line: the data in this paper are useful, and the authors are entitled to their biases about which structures are meaningful. This reviewer believes that the assembly description in the central paragraph on p.12 is probably wrong, but that should not prevent publication. Authors who publish solid data (and this laboratory certainly qualifies) have earned the right to speculate a bit. My recommendation is to simplify the narrative, try to avoid relying on supplementary figures (most readers haven't the patience to look at them), and reduce the posturing about the one-step protocol, which is a very helpful addition to the methods described in the literature, but does bring with it the bias that association with Ame1 defines what will be most interesting.

Response: We appreciate the feedback from this reviewer and have made the following changes to manuscript in response:

a. We have revised the manuscript to avoid implying that earlier structures are incorrect and instead point out that our technique is complementary. We agree that current structures as well as our own work may contain assembly biases and our intention is to provide additional native snapshots, not a definitive in vivo pathway.

b. We have reduced the supplementary material by eliminating apo-CCAN comparison and moved some into the main text (Fig.2a-b).

c. We have eliminated a step-wise model cartoon and made it clear the proposed assembly ideas are just models.

NOTE: I was unable to download the "related MS", so if it contains information that would answer some of my questions or contradict some of my points, feel free to ignore those items.

Reviewer #2 (Remarks to the Author)

In the manuscript “Native yeast kinetochore structures identify an essential inner kinetochore interaction,” Jiang et al. describe several cryoEM structures of centromeric complexes from S. cerevisiae obtained directly from yeast. The authors lysed mitotically arrested yeast, digested the DNA, and subjected the lysate to a sucrose gradient. Centromeric components were enriched by immunoprecipitation using FLAG-Ame1 protein, and the pulled complexes were analyzed by cryoEM. In a parallel experiment, before immunoprecipitation, the authors added 500 bp DNA containing a specific yeast centromeric sequence and performed another set of cryoEM analyses on the

assembled complexes, referring to these as “assembly IP.”
In the IP without added DNA, the authors detected only the monomeric CCAN complex, which closely resembled the high-resolution structure previously reported using purified components. However, in the IP with added DNA, the authors observed a CCAN dimer with a different orientation than that reported for purified CCAN components. Interestingly, they did not see DNA in these complexes, although they concluded that DNA is necessary for assembly, as the dimer is absent in the IP without added DNA. They also observed Cbf1-CCAN bound to the CEN sequence with more pronouncedly bent DNA.
Most notably, the CBF3 structure bound to CEN DNA revealed an extra density that the authors attribute to a trimer of the D3 domain of Ndc10. Previous literature had not shown that this part of Ndc10 could be involved in centromeric complex formation or that it directs trimerization (dimerization had been proposed previously). The authors used photobleaching, AF3, and mass photometry on purified Ndc10 D3 segment to confirm that the trimer observed in cryoEM is genuine.
To confirm the importance of the newly identified Ndc10 D3 trimer domain, they mutated six residues involved in its interaction with the Ndc10 D2 domain to alanines and found impaired yeast growth (in Mif2 conditional mutants) and reduced complex assembly in their IPs. Since they propose that the interaction of the D3 trimer with DNA stabilizes and bends DNA, they used Fiber-seq to assess DNA protection in assemblies made with wild-type and mutant Ndc10, finding that the wild type indeed protects more DNA. Finally, to test the importance of trimerization itself, they further mutated four hydrophobic residues involved in stabilizing the Ndc10 D3 trimer to alanines or aspartic acids and observed that these mutations do not interfere with in vitro complex assembly but result in yeast that cannot properly segregate chromosomes. Based on their structures, which they interpret as different steps in kinetochore assembly, and AF3 modeling, they propose a new sequence of events during kinetochore assembly.

This is very thorough and comprehensive work that reveals new and important aspects of yeast kinetochore assembly. All experiments are conducted to the highest standards, and interpretation, bias and future steps are properly addressed and discussed throughout the paper.

Minor comments for authors to consider:
- Include micrographs (with dimension bar) in the supplementary material.

Response: We have added micrographs with scale bars to Supplementary Fig. 1d.

- It would be helpful to know if the authors have tried using any other DNA in their assemblies, in terms of sequence or length.

Response: We used two lengths of CEN DNA to perform the assembly-IP. In the paper, we used a 500 bp template of CEN3 flanked by DNA. We have now compared this to a 250 bp template of CEN3 flanked by DNA. In this experiment, we used lysate that expresses Ame1-V5 and performed a standard assembly then IP through V5 conjugated beads. We didn't see significant difference in terms of the kinetochore

proteins pulled down between the two DNA length and therefore did not add this data to the paper.

- Pg 8: Supplementary figure 6e should be Supplementary figure 6f.

Response: We appreciate the reviewer noticing this mistake and we have corrected it in the revised manuscript.

Reviewer #3 (Remarks to the Author)

I co-reviewed this manuscript with one of the reviewers who provided the listed reports. This is part of the Nature Communications initiative to facilitate training in peer review and to provide appropriate recognition for Early Career Researchers who co-review manuscripts.

Reviewer #4 (Remarks to the Author):

The kinetochore is an important structure that forms on the centromere to ensure the faithful segregation of chromosomes. To understand its structure, many studies have been performed using recombinant proteins and in vitro reconstituted complexes. While this structural information is useful, much remains unknown about how the kinetochore is organized in cells. In this paper, the authors address the challenge of understanding the structure of the kinetochore in budding yeast cells. They developed a new method to purify the native inner kinetochore complex. Using this method, the authors purified several complexes and applied them to cryo-EM analysis. They identified four distinct inner kinetochore complexes, two of which had not been previously visualized. They discovered that Ndc10 forms a trimer that associates with centromeric DNA and the CBF3 complex. Using yeast genetics, the authors demonstrated that Ndc10 trimerization is essential for kinetochore formation.

Overall, this is an interesting observation. They identified native kinetochore complexes using a newly developed method. This method is very useful. It is also notable that they found differences in the structures of native complexes from reported structures using reconstituted complexes. Notably, Ndc10 trimerization was absent from the reconstituted complexes. In addition to structural data, they conducted comprehensive studies to reach their conclusions. The data quality is relatively high, and the manuscript is suitable for publication in Nature Communications. I raised several points in the hope that they would be useful for improving the paper's quality further.

1. Although the authors ultimately focused on Ndc10 trimerization, they made other interesting observations. For example, the structure of the CCAN dimer is interesting,

and I am curious about its biological significance. Based on their interpretation in the paper, the interaction between Mif2 (343-363) and Okp1 (132-152) may be involved in the formation of the CCAN dimer in coordination with DNA. I am uncertain about such a structure. It would be helpful if the authors proposed a structural model using AF3. Additionally, it would be helpful if they could show mutant data for Mif2 (343-363) or Okp1 (132-152).

Response: Reviewer 1 also questioned the relevance of the CCAN dimer. Although we do not know its function, we did make a mutant to abolish the dimer and it exhibits a genetic interaction with *mif2-3* (see reviewer 1, second comment 2). We have added this assay result to Supplementary Fig. 3g-i since it suggests the dimer contributes to inner kinetochore function.

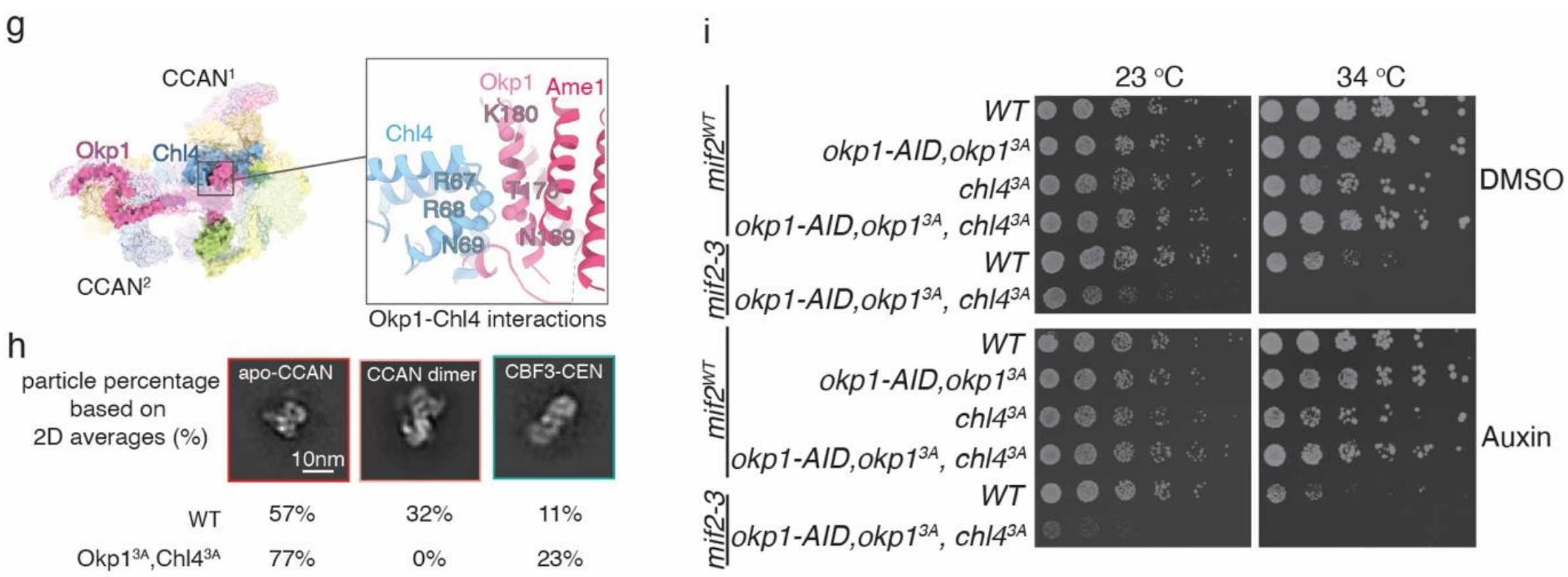

As requested, we also made the Mif2 (343-363 deleted) and Okp1 (132-152 deleted) mutants. We expressed the mutants in cells containing *mif2-AID* or *okp1-AID* and performed viability assays on both mutants. We observed a slight growth defect in the okp1 mutant strains at 37 ºC or in the presence of benomyl (see images below). Because reviewer 1 asked us to remove data, we did not include this additional data in the paper.

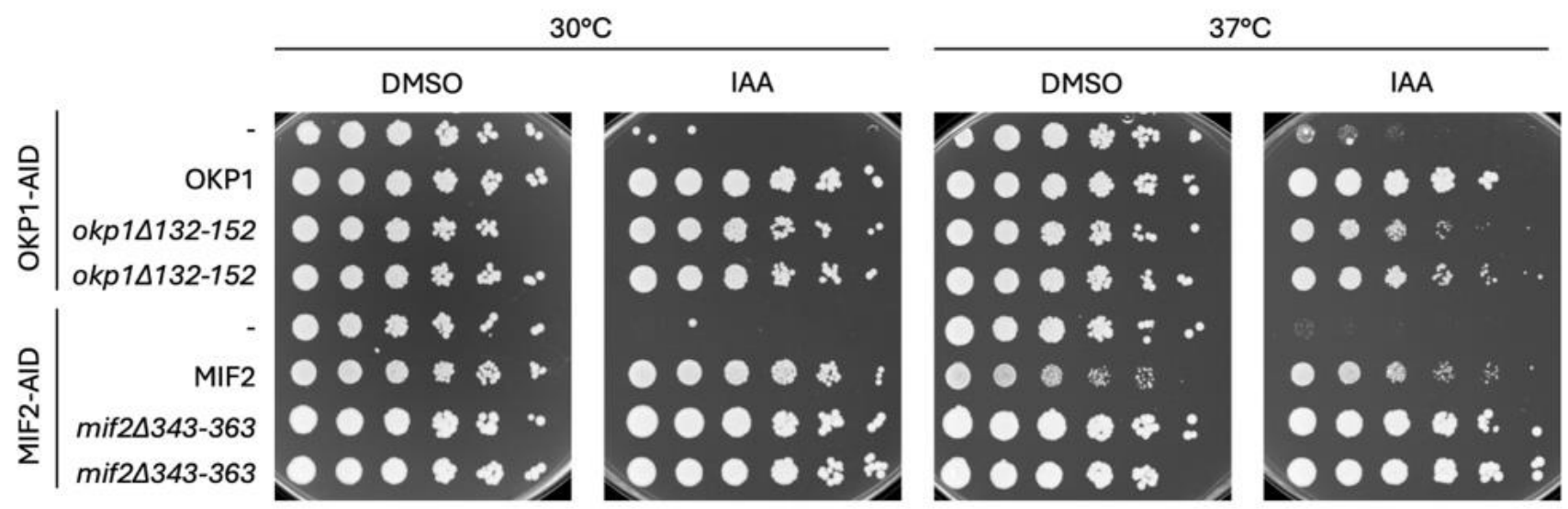

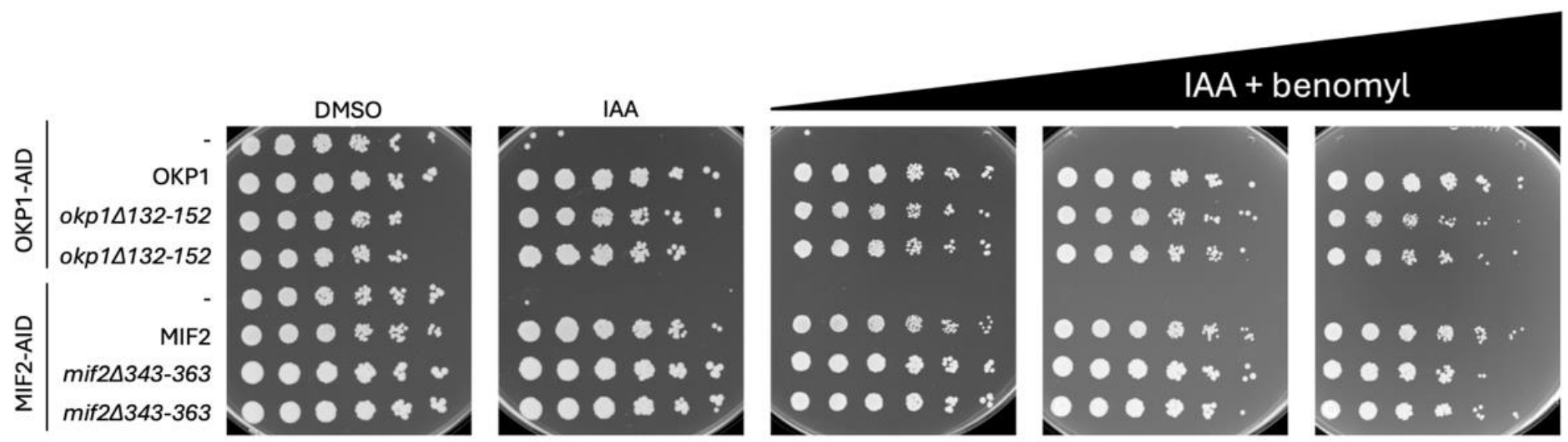

We also used AlphaFold 3 to predict the possible interaction between full-length Mif2 and Okp1. Even though the output 5 structures vary, we found that one of the structures resembled our speculation and have now reported this in Supplementary Fig. 3e-f.

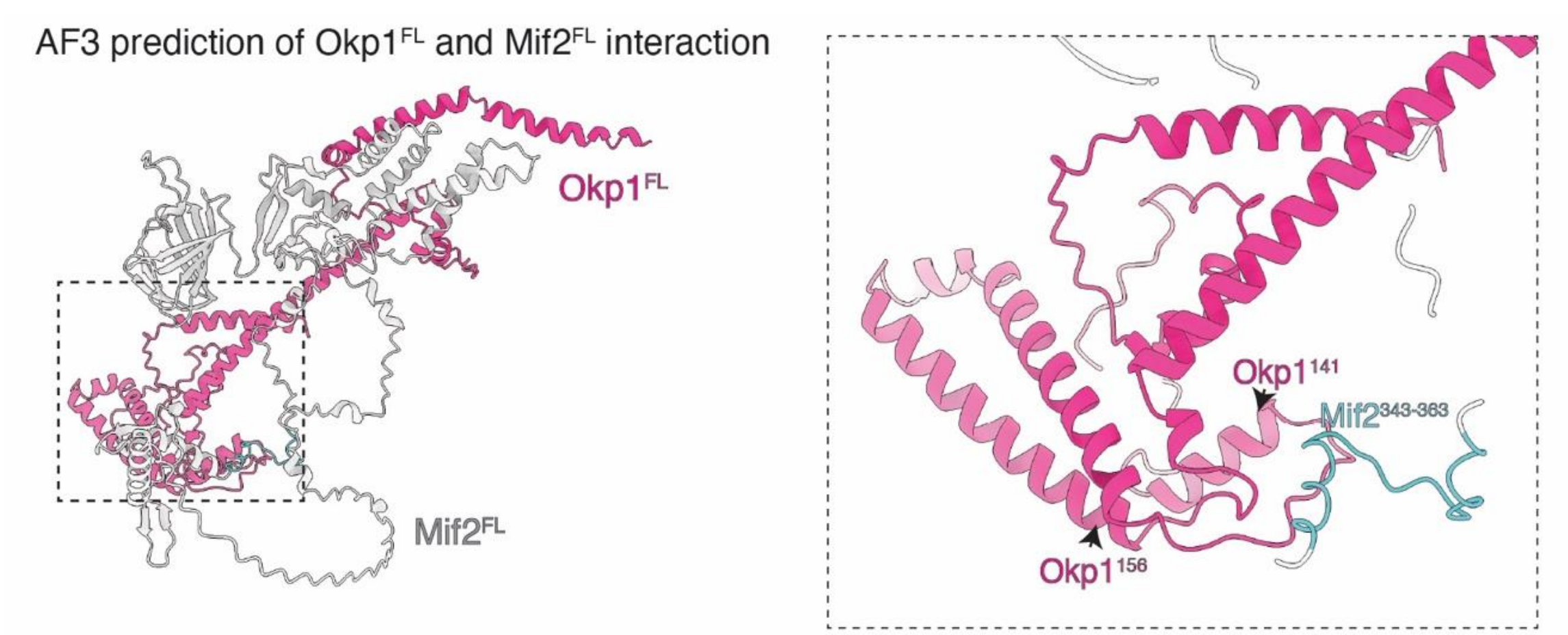

2. The authors propose that the binding of CBF3 or Ndc10 to DNA facilitates centromeric nucleosome assembly by bending the centromeric DNA. Although this is an attractive model, I feel it is a slight overstatement. I am unsure if they have data showing that mutations in the DNA-binding sites of the CBF3 complex or Ndc10 cause assembly defects in the Cse4 nucleosome. If they had such data, it would support the model. If not, however, they should tone down the model. I am also curious about the

cellular phenotype of yeast with Ndc10 two lysine mutations and loop 194-208 mutations (DNA-binding mutations).

Response: Reviewer 1 also pointed out that we should not propose that bending the centromeric DNA is necessary for nucleosome assembly. We have therefore removed this idea from the manuscript and instead suggest that the bending we observe may facilitate nucleosome formation and stabilize the nucleosome. Overall, we have toned down the comments about bending throughout the manuscript.

We did not attempt to make the Ndc10 mutants because any disruption of Ndc10 localization will cause a nucleosome assembly defect due to a lack of Scm3 chaperone localization to the centromere. These mutants will not allow us to separate the role of Ndc10 in any potential DNA bending role versus its function in chaperone-mediated nucleosome assembly so we did not make these mutants.

3. Related to comment #2. They could not observe Cse4 in their structures. This may be due to the flexible structure of the Cse4 nucleosome in cells. However, since they cannot see Cse4 in their structure, the final model in Figure 5H is too strong.

Response: We have eliminated the model in Figure 5h and instead show possible intermediate assemblages but no longer suggest we have identified a pathway to nucleosome formation (see discussion).

4. The Ndc10 (6A) mutant is interesting because it shows synthetic lethality with an Mif2 mutant. However, there is no evidence that the mutant exhibits binding defects with another Nud10. It would be good if they showed some data for this.

Response: We have clarified our writing about this interaction. The Ndc10 trimer docks onto CBF3 by interacting with the domain 2 of one of the Ndc10 proteins that is within the trimer. The mutant that we made is predicted to disrupt this interaction but the trimerization will not be affected so the three Ndc10 proteins will still interact. Therefore, we don't have a way to easily demonstrate that the mutant disrupted the interaction. Given that the fiber-seq data on this mutant shows that there is a decreased protection of part of the centromere, we think it is likely this is consistent with the mutant disrupting the interaction.

5. Regarding comment #4, the existence of Ndc10 in cells is unclear (Figure 5h). Figure 5h shows the Ndc10 trimer domain. It is assumed that full Ndc10 forms a trimer via the trimer domain. Does this mean that if the Ndc10 trimer binds to another Ndc10, it may form a tetramer? Please clarify this point. It would be better if they showed some suggestive data for tetramer formation in cells.

Response: We did not mean to imply that Ndc10 forms a tetramer. We have clarified the writing about this interaction to emphasize that the interaction between the trimer and CBF3 is mediated through the D2 domain of an Ndc10 protein that is in the trimer and not an additional Ndc10 protein that would imply a tetramer.

References

1. Popchock, A. R. *et al.* Stable centromere association of the yeast histone variant Cse4 requires its essential N-terminal domain. *EMBO J.* **44**, 1488–1511 (2025).
2. Joglekar, A. P., Salmon, E. D. & Bloom, K. S. Counting kinetochore protein numbers in budding yeast using genetically encoded fluorescent proteins. in *Methods in Cell Biology* vol. 85 127–151 (Elsevier, 2008).
3. Cieslinski, K. *et al.* Nanoscale structural organization and stoichiometry of the budding yeast kinetochore. *J. Cell Biol.* **222**, e202209094 (2023).
4. Dendooven, T. *et al.* Cryo-EM structure of the complete inner kinetochore of the budding yeast point centromere. *Sci. Adv.* **9**, eadg7480 (2023).

We have addressed the comments as described below:

REVIEWERS' COMMENTS

Reviewer #1 (Remarks to the Author):

The revised MS has met most of my concerns. While my own intuition is that DNA bending is not a "big deal" and that the function of the Ndc10 oligomerization element (clearly trimerization here) has to do with other aspects of the early assembly pathway, I have no objection to the speculations offerred by Jiang et al.

Whle reading carefully the revised version, I realized that there is a question I should have raised earlier (and that should NOT delay acceptance and publication). The second CBF3core, seen here and in 6GYS, could easily be an in vitro addition, recruited by Ndc10b (and by the extra DNA). In the (admittedly very early, with less fancy methods that we now have) crosslinking and footprinting work of Espelin et al, there was evidence of additional Ndc10 to the "right" of the well-bound two, but no evidence of an additional CBF3 core. Likewise, the fluorescence counting numbers from Joglekar indicate one, not two CBF3 cores per kinetochore (one Cep3 dimer), but more than one Ndc10 dimer (consistent with a single trimer, although the number in their metaphase occupancy table was 4, not 3). The present authors mention (almost parenthetically) the possibility of an "experimental artifact" yielding the second CBF3 core, but another possibility is that it is a transient occupancy of the early intermediates studied here.

Response: We appreciate the thoughtful idea from the reviewer. We have added this additional idea after our comment about a possible experimental artifact:

We also do not know if a second CBF3 complex on periCEN DNA is an experimental artifact, although our Fiber-seq data suggests there is likely a second copy of CBF3 instead of CCAN in this region. One possibility is that there is a transient intermediate where a second copy of CBF3 associates with the pericentromeric DNA during an early step of kinetochore assembly.

Reviewer #4 (Remarks to the Author):

This is a revised version of the manuscript. They found that Ndc10 forms a trimer that binds to centromeric DNA and the CBF3 complex. While I thought the previous version was well-written, the paper has improved with the addition of data and revisions. I believe the authors addressed the reviewers' concerns. Therefore, I recommend accepting this manuscript for publication.

Response: We are grateful to the reviewer for their support of our work.
